# Contribution of Hypothyroidism to Cognitive Impairment and Hippocampal Synaptic Plasticity Regulation in an Animal Model of Depression

**DOI:** 10.3390/ijms22041599

**Published:** 2021-02-05

**Authors:** Katarzyna Głombik, Jan Detka, Bartosz Bobula, Joanna Bąk, Magdalena Kusek, Krzysztof Tokarski, Bogusława Budziszewska

**Affiliations:** 1 Laboratory of Immunoendocrinology, Department of Experimental Neuroendocrinology, Maj Institute of Pharmacology, Polish Academy of Sciences, Smętna 12, 31-343 Kraków, Poland; detka@if-pan.krakow.pl (J.D.); budzisz@if-pan.krakow.pl (B.B.); 2 Department of Physiology, Maj Institute of Pharmacology, Polish Academy of Sciences, Smętna 12, 31-343 Kraków, Poland; bobula@if-pan.krakow.pl (B.B.); j.sowa@if-pan.krakow.pl (J.B.); kusek@if-pan.krakow.pl (M.K.); ktok@if-pan.krakow.pl (K.T.)

**Keywords:** depression, hypothyroidism, synaptic plasticity, cognition

## Abstract

The role that thyroid hormone deficiency plays in depression and synaptic plasticity in adults has only begun to be elucidated. This paper analyzes the possible link between depression and hypothyroidism in cognitive function alterations, using Wistar–Kyoto (WKY—an animal model of depression) rats and control Wistar rats under standard and thyroid hormone deficiency conditions (propylthiouracil administration—PTU). A weakening of memory processes in the WKY rats is shown behaviorally, and in the reduction of long-term potentiation (LTP) in the dentate gyrus (DG) and CA1 hippocampal regions. PTU administration decreased LTP and increased basal excitatory transmission in the DG in Wistar rats. A decrease in short-term synaptic plasticity is shown by the paired-pulse ratio measurement, occurring during hypothyroidism in DG and CA1 in WKY rats. Differences between the strains may result from decreases in the p-CaMKII, p-AKT, and the level of acetylcholine, while in the case of the co-occurrence of depression and hypothyroidism, an increase in the p-ERK1-MAP seemed to be important. Obtained results show that thyroid hormones are less involved in the inhibition of glutamate release and/or excitability of the postsynaptic neurons in WKY rats, which may indicate a lower sensitivity of the hippocampus to the action of thyroid hormones in depression.

## 1. Introduction

Although extensive research has been performed, the etiology of depressive disorder is not fully understood, and therefore, the currently available antidepressant drugs are not effective in some patients. One of the approaches to the therapy of treatment-resistant depression is supportive treatment with thyroid hormones (THs) [1]; however, the mechanism of action and the roles of these hormones in the pathogenesis of depression are not known. This is mainly because the effect of thyroid hormones in the central nervous system in adults is not well-studied. It is well established that thyroid hormones regulate many important processes, such as growth, differentiation, migration and neuronal integration, glial cell proliferation, myelination, and neurotransmitter synthesis during development, but the vast majority of the genes regulated by these hormones become insensitive after the period of brain development. It is known that proper TH function during pregnancy is crucial for the development of the central nervous system in the fetus, and under-activity of the thyroid gland can lead to mental dysfunction and impairments in cognitive development [2]. The present, although still very fragmentary, data [3,4] indicate that even in the adult brain, thyroid hormone deficiency impairs certain functions (mood, memory, and learning processes). We can assume that this impairment is probably due to thyroid hormone-mediated interference in metabolic processes and intracellular signaling pathways. In the adult brain, various genetic loci that are responsible for thyroid hormones have been identified; moreover, these hormones act not only on genes that contain thyroid response elements (TREs), but also on other genes and by nongenomic mechanisms [5]. The participation of TH in the course of depression is evidenced by epidemiological data showing that 1–4% of patients with the affective disorder suffer from hypothyroidism, and approximately 4–40% show symptoms of subclinical hypothyroidism. Furthermore, in people with depression, abnormalities of the thyroxine (T4) to triiodothyronine (T3) ratio, elevated reverse T3 levels, blunted TSH response to TRH, and the presence of anti-thyroid antibodies are observed more often than in the healthy population [5,6]. Some studies suggest that in depression, there may be a reduction in the activity, but not necessarily the level of thyroid hormones. This is shown by clinical observations indicating that effective therapy of depression with T4 requires higher doses than those used in the treatment of primary thyroid disorders, and additionally, in depressed patients, supraphysiological doses of T4 cause fewer side effects than in healthy people [7]. Moreover, the levels of thyroid hormones in the brain do not correlate with their peripheral concentrations, because only approximately 20% of T3, the active form of thyroid hormone in the brain, comes from blood, whereas most is produced in glial cells from T4. Additionally, thyroid hormone content in particular brain regions depends on the expression of their transporters and deiodinases [8].

The involvement of thyroid hormones in the pathogenesis of depression is also indicated by some symptoms observed in both depression and hypothyroidism. Cognitive function impairment is a characteristic and frequently observed manifestation in both of these diseases. For example, clinical studies reported severe cognitive disabilities, including an inability to concentrate, calculate and understand complex questions, slow mentation, and weakened memory for recent events during the course of hypothyroidism [9]. Experimental studies have demonstrated that hypothyroidism impairs learning, short-term and long-term memory, induces changes in neurotransmitters and signaling molecules, and disrupts synaptic plasticity [10,11]. As we have shown in previous studies, thyroid hormone deficiency also leads to metabolic disturbances in the brain both in control animals and in a depression model [12].

In light of current knowledge, the present study aimed to analyze the possible link between depression and thyroid hormone deficiency and to explore the mechanisms that underlie cognitive impairment in depression in adulthood, considering dysregulation of thyroid endocrine homeostasis. For this purpose, we performed this study in a model of endogenous depression, the Wistar–Kyoto (WKY) rats, in comparison to control Wistar rats under standard and thyroid hormone deficiency conditions. Propylthiouracil (PTU, an anti-thyroid agent) has been used to induce hypothyroidism in both strains. In the present work, we assessed the behavioral status of the examined animals with the use of tests to measure spatial memory and anxiety-like behavior and evaluated memory formation processes in an electrophysiological study by measuring synaptic long-term potentiation (LTP), short-term synaptic plasticity (paired-pulse responses) and basal excitatory transmission. We also examined the potential molecular mechanisms that could be responsible for the changes in the measured electrophysiological parameters in the studied models of depression and hypothyroidism. To achieve this, we evaluated factors involved in the regulation of cognitive functions that may be affected by both depression and hypothyroidism, such as acetylcholine, synaptotagmin 1, NMDA (*N*-methyl-d-aspartate) receptor subunits, mitochondrial fusion and fission markers, enzymes regulating glycogen levels, growth factors, caspase-1, and protein kinases essential for the induction and/or maintenance of LTP (Calcium/calmodulin-dependent protein kinase II isoform K (CaMKII), Extracellular signal-regulated kinases 1 and 2 (ERK1 and 2), cAMP Response Element-Binding Protein (CREB), and protein kinase B (AKT1)). We also examined whether the changes observed in depression or hypothyroidism are exacerbated in the coexistence of these two diseases.

## 2. Results

### 2.1. Behavioral Study

#### 2.1.1. Anxiety Behavior Evaluated in the Elevated Plus Maze (EPM) Test

**Figure 1:**
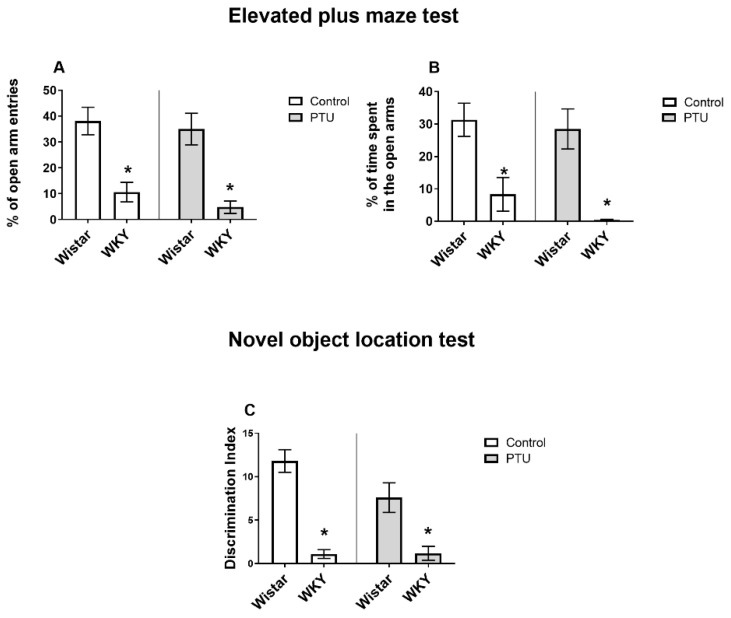
The effects of strain and propylthiouracil (PTU) treatment on the % of open arm entries (**A**) and % of time spent in the open arms (**B**) measured in the elevated plus maze test and on the object discrimination index (**C**) measured in the novel object location test. The results are expressed as the mean ± SEM. * *p* < 0.05 vs. the control group (Wistar rats). *n* = 8–10.

In the EPM test, a decrease in the percentage of open arm entries was observed in the WKY rats (strain effect: F_1,36_ = 39.40, *p* < 0.05) (Figure 1A). In addition, the percentage of time spent in the open arms was diminished in the WKY group than in the Wistar group (strain effect: F_1,36_ = 28.60, *p* < 0.05) (Figure 1B). PTU had no impact on these two parameters.

#### 2.1.2. Spatial Memory Evaluated in the Novel Object Location (NOL) Test

The WKY rats exhibited a lower object discrimination index than the Wistar rats (strain effect: F_1,33_ = 49.80, *p* < 0.05) (Figure 1C). PTU had no effect on spatial memory as measured in this test.

### 2.2. Electrophysiological Study

#### 2.2.1. The Effects of PTU on Field Potentials (FPs) in the CA1 Region of the Hippocampus in Wistar and WKY Rats

**Figure 2:**
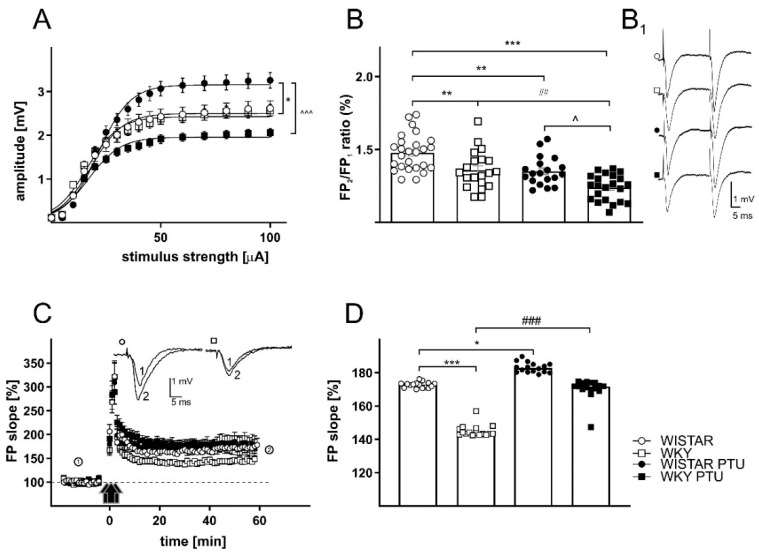
The effects of strain and PTU treatment (filled symbols) on the relationship between the stimulus intensity and amplitude of field potentials (**A**), paired-pulse ratio (**B**), examples of individual field potentials (FPs) evoked by paired stimuli (**B_1_**) and long-term potentiation (LTP) recorded in the CA1 hippocampal area of Wistar (circles) and WKY (squares) rats (**C**,**D**). The graph shows plots of the mean amplitudes ± SEM (**C**). Effects of PTU on LTP induced by high-frequency stimulation (HFS) in hippocampal CA1 areas in slices prepared from Wistar rats (filled circles) and WKY rats (filled squares). The insets show examples of FP slopes recorded at the indicated times before (1) and after (2) PTU administration. The mean slopes of FPs recorded between 45 and 60 min after HFS in control slices, and slices prepared from PTU-treated animals are shown. The results are expressed as the mean ± SEM. * *p* < 0.05 vs. the control group (Wistar rats), ** *p* < 0.01 vs. the control group (Wistar rats), *** *p* < 0.001 vs. the control group (Wistar rats), ## *p* < 0.01 vs. the WKY, ### *p* < 0.001 vs. the WKY group, ^ *p* < 0.05 vs. Wistar PTU group, ^^^ *p* < 0.001 vs. Wistar PTU group. *n* = 6–8.

The relationship between stimulus intensity and field potential (FP) amplitude (input-output curve) was determined with a stimulation intensity range of 0–100 µA. There were significant effect of strain · treatment interaction (F_1,86_ = 13.07, *p* < 0.001) on FP amplitude in the CA1 hippocampal area. The maximum field potential amplitude (Vmax) measured in the CA1 region of the hippocampus in brain slices prepared from the control Wistar rats was 2.50 mV, which did not differ significantly from that observed in slices from the WKY rats (2.42 mV). In the Wistar rats receiving PTU, the maximum amplitude was significantly higher than that in the control animals (3.15 mV vs. 2.50 mV, respectively; *p* < 0.05), but there were no changes in the parameter characterizing the slope of the curve. In the WKY rats, administration of PTU did not change the amplitudes of FPs (2.42 vs. 1.95 mV) and did not affect the slope of the curve; however, the WKY rats treated with PTU had significantly lower FP amplitudes than the Wistar rats with hypothyroidism (*p* < 0.001; Table 1, Figure 2A).

#### 2.2.2. The Effects of PTU on Field Potentials (FPs) in the Dentate Gyrus (DG) Region of the Hippocampus in Wistar and WKY Rats

**Figure 3:**
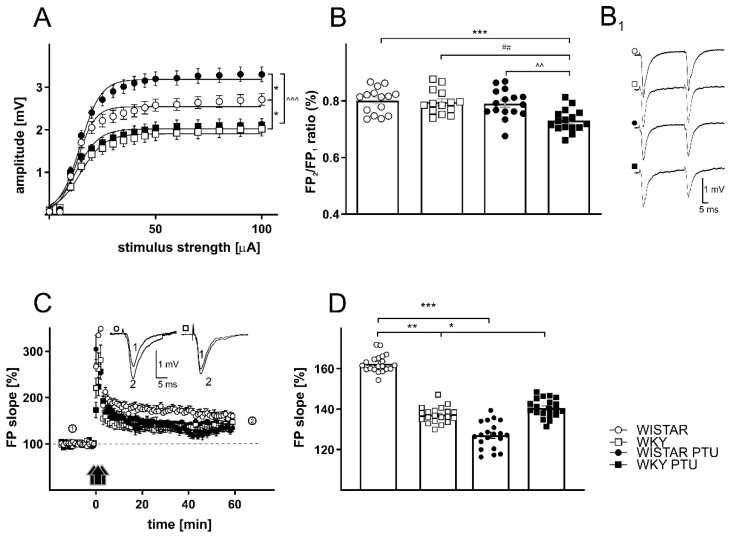
The effects of strain and PTU treatment (filled symbols) on the relationship between stimulus intensity and the amplitude of field potentials (**A**), paired-pulse ratio (**B**), examples of individual FPs evoked by paired stimuli (**B_1_**) and LTP recorded in the DG hippocampal area of Wistar (circles) and WKY (squares) rats (**C**,**D**). The graph shows plots of the mean amplitudes ± SEM (**C**). Effects of PTU on LTP induced by HFS stimulation in hippocampal DG areas in slices prepared from Wistar rats (filled circles) and WKY rats (filled squares). The insets show examples of FP slopes recorded at the indicated times before (1) and after (2) PTU administration. The mean slopes of FPs recorded between 45 and 60 min after HFS in control slices, and slices prepared from PTU-treated animals are shown. The results are expressed as the mean ± SEM. * *p* < 0.05 vs. the control group (Wistar rats), ** *p* < 0.01 vs. the control group (Wistar rats), *** *p* < 0.001 vs. the control group (Wistar rats), ## *p* < 0.01 vs. the WKY, ^^ *p* < 0.01 vs. Wistar PTU group, ^^^ *p* < 0.001 vs. Wistar PTU group. *n* = 6–8.

There was a significant effect of strain (F_1,71_ = 31.60, *p* < 0.001) and treatment (F_1,71_ = 5.202, *p* < 0.05) on FP amplitude in the dentate gyrus of the hippocampus. Treatment with PTU significantly increased the amplitudes of FPs from 2.54 to 3.18 mV in the Wistar rats (*p* < 0.05) without changing the parameter characterizing the slope of the curve. In the WKY rats, PTU administration had no effect on the amplitudes of FPs (1.90 mV in the control WKY rats; 2.02 mV in WKY rats with PTU). There was a significant difference in the amplitudes of FPs of the Wistar and WKY rats without PTU (*p* < 0.05), and the amplitudes of FPs were significantly lower in the WKY rats administered PTU than in the Wistar rats with hypothyroidism (*p* < 0.001; Table 2, Figure 3A).

#### 2.2.3. The Effects of PTU on Paired-Pulse Ratio (PPR)

Paired-pulse stimulation, a model of short-term synaptic plasticity, was induced with two stimuli of equal intensity (approximately 30% of maximum) at a time interval of 50 ms. Facilitation or inhibition was measured as the ratio of the fEPSP slope of the second stimulus to that of the first stimulus averaged over three responses per pulse pair. In the CA1 region, we found an effect of strain (F_1,83_ = 21.97 *p* < 0.001), as well as treatment (F_1,83_ = 27.02, *p* < 0.001) on the PPR. The PPR measured in the WKY rats was lower than that measured in the Wistar rats (*p* < 0.01). PTU treatment resulted in a significant decrease in PPR in the CA1 region in the Wistar (*p* < 0.01) and WKY rats (*p* < 0.01; Table 3, Figure 2B). There was also a significant decrease in the PPR between the WKY PTU and Wistar PTU rats (*p* < 0.05, Table 3, Figure 2B). It was found that in the DG PPR changes were connected with treatment (F_1,57_ = 10.97, *p* < 0.01) and strain · treatment interaction (F_1,57_ = 5.582, *p* < 0.05). Moreover, the PPR measured in the DG did not differ between the WKY and Wistar rats. Administration of PTU did not affect this parameter in the Wistar rats, whereas in the WKY rats treated with PTU, a significant decrease in the PPR ratio compared to that of the WKY rats was observed (*p* < 0.01). Moreover, there was a significant decrease in the PPR between the WKY PTU and Wistar PTU rats (*p* < 0.01, Table 3, Figure 3B).

#### 2.2.4. The Effects of PTU on Long-Term Potentiation

There were significant effects of strain (F_1,86_ = 17.93, *p* < 0.001) and treatment (F_1,86_ = 41.35, *p* < 0.001) on LTP measured in the hippocampal CA1 region. The LTP measured in the CA1 region of the hippocampus in brain slices prepared from the WKY rats was strongly reduced compared to that in slices from the Wistar rats (*p* < 0.001). Administration of PTU led to an increase in LTP in both rat strains in Wistar (*p* < 0.05) and in WKY (*p* < 0.001) (Table 4, Figure 2C,D).

In the dentate gyrus, a significant decrease in LTP was recorded in all study groups compared to the control Wistar group. The decrease was connected with the strain effect (F_1,71_ = 31.60, *p* < 0.001) and treatment (F_1,71_ = 5.202, *p* < 0.05), but no interaction was detected. LTP attenuation was observed in the WKY rats compared to the Wistar rats (*p* < 0.01). Administration of PTU reduced LTP in the Wistar rats (*p* < 0.001), but did not affect this parameter in the WKY rats (Table 4, Figure 3C,D).

### 2.3. Biochemical Study

#### 2.3.1. Gene Expression of Syt1, Opa1, Gys1, Dnm1l, Pygb, Bdnf, and Ngf

**Figure 4:**
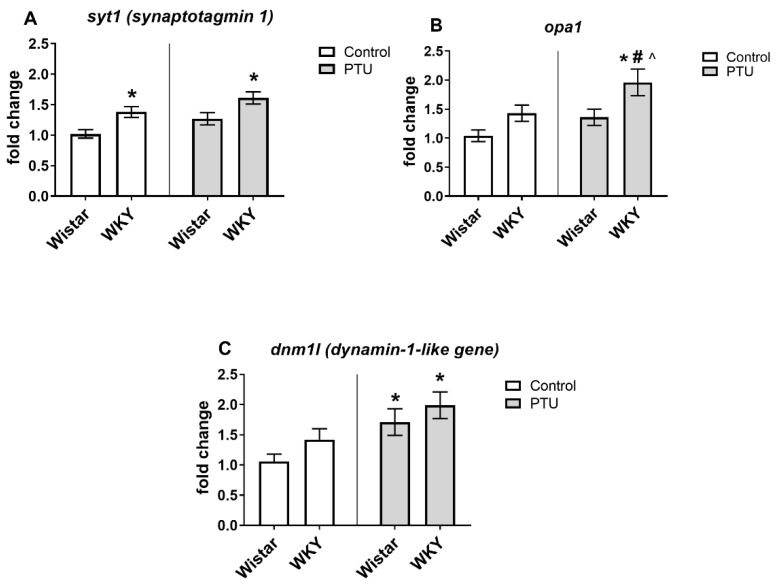
The effects of strain and PTU treatment on the gene expression of synaptotagmin 1 (syt1) (**A**), optic atrophy 1 (opa1) (**B**), and the dynamin 1-like gene (dnm1l) (**C**) in the hippocampus. The results are expressed as the average fold change ± SEM. * *p* < 0.05 vs. the control group (Wistar rats); # *p* < 0.05 vs. the WKY group; ^ *p* < 0.05 vs. Wistar PTU group. *n* = 9–10.

**Figure 5:**
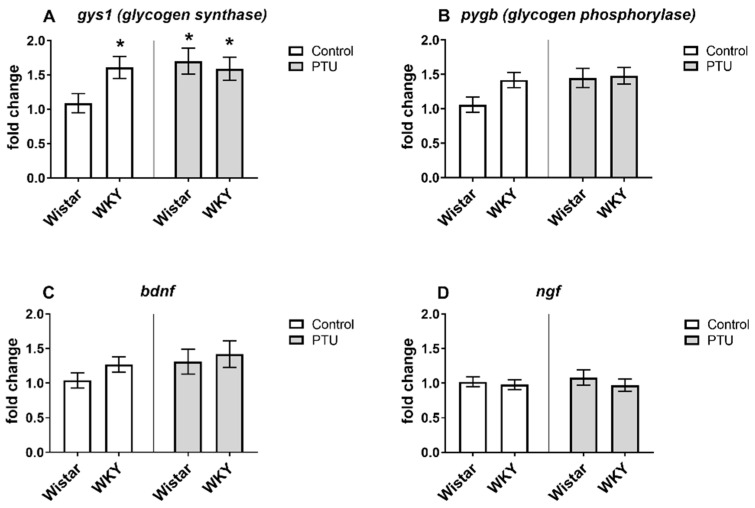
The effects of strain and PTU treatment on the gene expression of glycogen synthase 1 (gys1) (**A**), glycogen phosphorylase (pygb) (**B**), brain-derived neurotrophic factor (bdnf) (**C**), and nerve growth factor (ngf) (**D**) in the hippocampus. The results are expressed as the average fold change ± SEM. * *p* < 0.05 vs. the control group (Wistar rats). *n* = 9–10.

The expression of syt1 was significantly increased in the WKY rats compared to the Wistar rats (strain effect: F_1,36_ = 16.45, *p* < 0.05) both in animals without and with PTU administration (Figure 4A). Expression of opa1 was higher in the WKY animals treated with PTU than in other groups (strain effect: F_1,35_ = 9.39, *p* < 0.05, treatment effect: F_1,35_ = 6.78, *p* < 0.05) (Figure 4B). An impact of PTU was observed in the case of dnm1l and gys1 (treatment effects: F_1,34_ = 9.29 and F_1,33_ = 4.16, respectively, *p* < 0.05) in both Wistar and WKY rats. PTU upregulated the gene expression of the measured factors (Figure 4C and Figure 5A). Moreover, a post hoc test revealed that gys1 expression was enhanced in the WKY animals compared to the control animals. We did not observe any changes between the groups for the pygb-, bdnf-, or ngf -coding genes (Figure 5B–D).

#### 2.3.2. Protein Level of Subunits of NMDA Receptor (GluN1, GluN2A, GluN2B)

The Western blot analysis showed no changes between the groups in any of the NMDA receptor subunits (Table 5).

#### 2.3.3. Protein Level of Calcium/Calmodulin-Dependent Protein Kinase II Isoform K II (CaMKII, Total and Phospho Form) and AKT1 (Total and Phospho (Ser473) Form)

**Figure 6:**
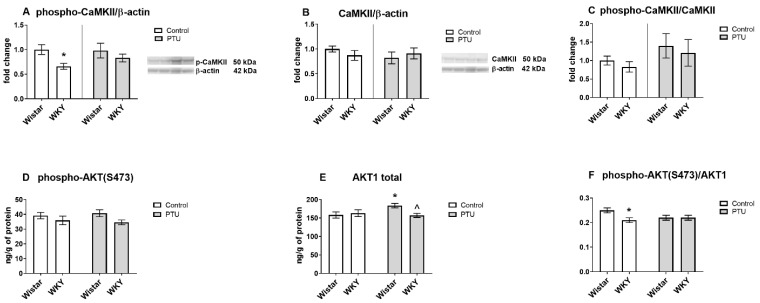
The effects of strain and PTU treatment on the protein levels of phospho-CaMKII (**A**), CaMKII (**B**), phospho-CaMKII/CaMKII (**C**), phospho(Ser473)-AKT1 (**D**), AKT1 (**E**) and phospho-AKT(S473)/AKT1 (**F**) in the hippocampus. The results are expressed as the average fold change ± SEM (**A**–**C**) or as the mean ± SEM (**D**–**F**). * *p* < 0.05 vs. the control group (Wistar rats); ^ vs. the Wistar PTU group. *n* = 8–10.

The phospho-CaMKII level was significantly lower in the WKY rats than in the control Wistar rats (strain effect: F_1,31_ = 5.52, *p* < 0.05), whereas the total CaMK II concentration did not differ between the tested rat strains and was not changed by PTU (Figure 6A,B). The ratio of phospho-CaMKII to the total form did not differ between the groups (Figure 6C). Phospho-AKT1 was not changed by either the strain or PTU (Figure 6D). An analysis of the hippocampal homogenates revealed increased levels of total AKT1 in the Wistar PTU-treated rats compared to those in the control rats and in the WKY rats that received PTU (strain · treatment interaction: F_1,34_ = 4.70, *p* < 0.05, Figure 6E). Additionally, the ratio phospho-AKT (Ser473)/total AKT1 was diminished in WKY rats (strain effect: F_1,34_ = 4.20, *p* < 0.05) (Figure 6F).

#### 2.3.4. Protein Level of CREB (Total and Phospho (Ser133) Form), Extracellular Signal-Regulated Kinases 1/2 (ERK1/2, Phospho and Total Form) 

**Figure 7:**
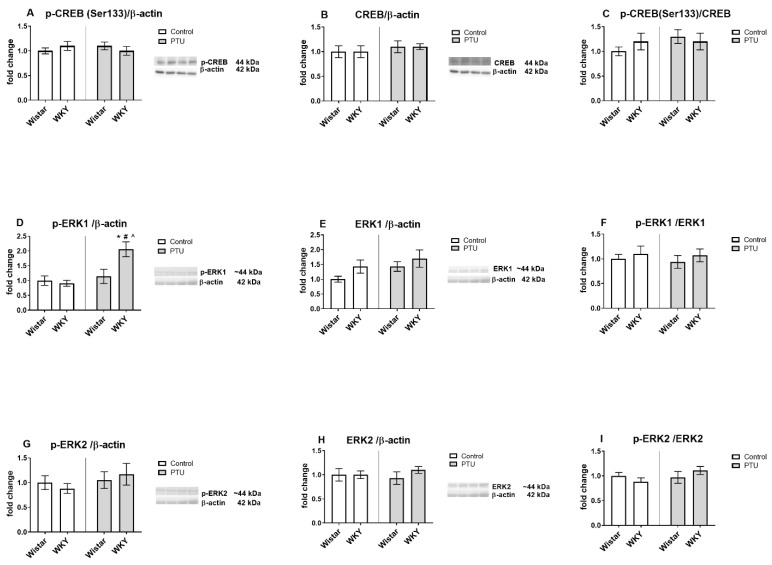
The effects of strain and PTU treatment on the protein levels of phospho-(Ser133) CREB (**A**), CREB (**B**), phospho-CREB (Ser133)/CREB (**C**), phospho-ERK1 (**D**), ERK1 (**E**), phospho-ERK1/ERK1 (**F**), phospho-ERK2 (**G**), ERK2 (**H**), and phospho-ERK2/ERK2 (**I**) in the hippocampus. The results are expressed as the average fold change ± SEM. * *p* < 0.05 vs. the control group (Wistar rats); # *p* < 0.05 vs. the WKY group, ^ vs. the Wistar PTU group. *n* = 8–10.

No changes in p-Ser133-CREB, total CREB protein levels, and their ratio were detected between the examined groups (Figure 7A–C). Regarding the levels of extracellular signal-regulated kinases, p-ERK1 was increased in the WKY rats treated with PTU compared to all other groups (strain effect: F_1,33_ = 4.43, strain · treatment interaction: F_1,34_ = 4.70, *p* < 0.05) (Figure 7D). The total form of ERK1 and p-ERK1/ERK1 ratio were not changed by the examined factors (Figure 7E,F). As shown in Figure 7G–I, the hippocampal levels of p-ERK2, total ERK2, and their ratio were not influenced by strain and PTU.

#### 2.3.5. Protein Level of Caspase-1 p20

**Figure 8:**
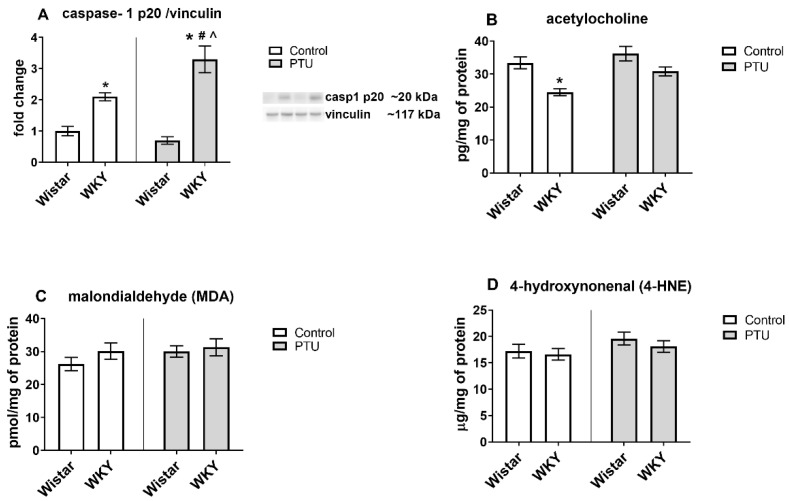
The effects of strain and PTU treatment on the protein levels of caspase-1 p20 (**A**), acetylcholine (**B**), malondialdehyde (MDA) (**C**), and 4-hydroxynonenal (4-HNE) (**D**). The results are expressed as the average fold change ± SEM (**A**) or the mean ± SEM (**B**–**D**). * *p* < 0.05 vs. the control group (Wistar rats); # *p* < 0.05 vs. the WKY group, ^ vs. the Wistar PTU group. *n* = 8–10.

The level of caspase-1 p20 was significantly upregulated in WKY rats (strain effect: F_1,32_ = 57.74, *p* < 0.05). Furthermore, PTU enhanced observed effect in this strain (strain · treatment interaction: F_1,32_ = 9.00, *p* < 0.05, Figure 8A). Moreover, an increased level of caspase-1 in WKY PTU rats in comparison to the Wistar PTU group was demonstrated.

#### 2.3.6. Acetylcholine Concentration

Significant differences between rat strains (strain effect: F_1,26_ = 7.87, *p* < 0.05), but no effect of PTU was observed in the acetylcholine levels in the hippocampal samples. The concentration of acetylcholine was significantly lower in the WKY rats than in the Wistar rats (Figure 8B).

#### 2.3.7. Protein Levels of Malondialdehyde (MDA) and 4-Hydroxynonenal (4-HNE)

The ELISA results showed no significant differences in either the MDA or 4-HNE hippocampal levels in the samples from any examined group (Figure 8C,D).

## 3. Discussion

The present study demonstrated anxiety-like behaviors, impairment of spatial memory, disturbances in electrophysiological parameters in the CA1 and DG hippocampal regions, and changes in some biochemical markers of neuronal plasticity in the hippocampus in rat models of depression, hypothyroidism, and the co-occurrence of depression and hypothyroidism.

In our investigation, WKY rats were used as a model of depression, since this rat strain exhibits a natural susceptibility to stress and is an established genetic model of endogenous depression [13,14,15,16]. As a control group, Wistar rats were used because these strains have similar genetic backgrounds, but differ in their susceptibility to stress [14,17]. Hypothyroidism was induced in the Wistar and WKY rats via administration of PTU in the drinking water for three weeks; as we have previously shown, this dosage lowers fT3 and fT4 and increases plasma TSH levels [12]. Since brain thyroid hormone levels do not correlate with their concentration in the blood, we previously determined the thyroid hormone content in the hippocampus and found that the level of T3 was decreased in Wistar rats receiving PTU and was even more substantially decreased in WKY rats [12].

The anxiety-like behavior examined in our study with the use of the elevated plus maze test is often observed in addition to depression-like behavior in various animal models of depression, including the WKY rats [18]. Abnormalities in learning and memory processes in WKY rats have been shown previously in the Morris water maze [19] and novel object recognition test [12]. These data are in agreement with our findings that spatial memory impairment, as assayed in the novel object location (NOL) test, also occurred in this rat strain. The behavioral changes indicating a weakening of memory processes in the WKY rats compared to those of the Wistar rats were supported by the electrophysiological recordings showing a reduction of LTP (a form of synaptic plasticity widely accepted as a cellular correlate of learning and memory) in the WKY rats. In contrast to the differences observed between the rat strains in both the NOL test and the LTP measurements, the induction of hypothyroidism did not show such a clear impact. LTP reduction was observed only in the dentate gyrus of the hippocampus in the Wistar rats receiving PTU, and only in rats of this strain was a downward trend in the discrimination index observed in the NOL test. Many data concern the adverse effects of thyroid hormone deficiency during the developmental stage on cognitive functions, but the impact of hypothyroidism on adult animals is poorly reported. Some papers have established that hypothyroidism also leads to LTP impairment in adult male Wistar rats. Similar to the results of our study, a decrease in LTP in the dentate gyrus of the hippocampus was previously observed in Wistar rats treated with PTU and in this rat strain after thyroidectomy [20,21,22]. However, contrary to our results, a decrease in LTP in the CA1 area was observed in Wistar rats after thyroidectomy [10,23]. Most likely, these differences result from the use of various methods to lower thyroid hormone levels (PTU administration vs. thyroidectomy) that differ in the intensity of reduction of the measured hormones. In the WKY rats, PTU did not affect LTP in the DG, which was initially lower in this strain; however, it was difficult to explain the observation that in the CA1 region of the hippocampus, PTU administration resulted in LTP intensification. As we did not measure LTD, it cannot be ruled out that despite the increase in LTP, the LTP/LTD ratio was reduced. Nevertheless, these results indicate that thyroid hormones have different effects on the LTP process in WKY and Wistar rats and that there may be other mechanisms of action of these hormones in the studied regions of the hippocampus.

As in the case of LTP, other effects of PTU on basal excitatory transmission were also observed in the WKY rats compared to the Wistar rats. Basal excitatory transmission, measured as the input-output relationship, was significantly increased in both hippocampal regions in slices obtained from the Wistar rats treated with PTU. The same effect of hypothyroidism was also shown in young male Sprague Dawley rats [24], while in the present study in WKY rats, which were used as a model of depression, PTU had no effect on the magnitude of fEPSPs. The increase in amplitude associated with the deficit of thyroid hormones in Wistar rats may result from presynaptic changes–for example, increased release of glutamate or postsynaptic changes–in the density or reactivity of postsynaptic receptors. The observation that inhibition of thyroid hormone synthesis had no effect on the amplitude of fEPSPs in WKY rats suggested that in this rat strain, thyroid hormones are less involved in the regulation of the glutamate release and/or excitability of postsynaptic neurons. In fact, some studies have shown changes in basal and induced glutamate levels, expression of NMDA receptor subunits, and NMDA receptor binding in WKY rats compared to Wistar rats [18]. In WKY rats, as well as in humans with treatment-resistant depression, central sensitivity to thyroid hormones seems to be reduced, which in turn may lead to the weakened or absent inhibition of glutamate release or the excitability of the postsynaptic receptors by these hormones [18,25].

Abnormalities in the mechanisms of presynaptic neurotransmitter release were also shown by changes in paired-pulse responses. PPR is a kind of short-term synaptic plasticity that could be dependent on presynaptic mechanisms of neurotransmitter release. The recorded changes in PPR may have been caused by residual calcium availability induced by previous stimulations and/or changes in inhibition mediated by GABAergic interneurons [26]. In the CA1 region of the hippocampus, PPR facilitation manifested as an increase in the amplitude of the second response, while in dentate gyrus cells, the amplitude of the second response was lower than that of the first. Paired-pulse data indicated that PTU administration affected presynaptic release mechanisms in the perforant path of DG synapses in the WKY rats and not in the Wistar rats because only the WKY rats showed a decrease in PPR after PTU. In contrast, Shaffer collateral-CA1 synapses were affected in both Wistar and WKY rats, as evidenced by the decreased PPR after PTU treatment. Moreover, the WKY rats displayed a reduced PPR compared to the Wistar rats under baseline conditions, suggesting that there are subtle differences in presynaptic release machinery between these two strains. The changes in PPR, particularly in the CA1 region of the hippocampus, indicated differences in the mechanisms of presynaptic neurotransmitter release between the Wistar and WKY rats and dysregulation of this process under decreased thyroid hormone conditions. The strongest PPR downregulation was shown in the animal model of the co-occurrence of depression and hypothyroidism, i.e., in the WKY rats treated with PTU. A similar reduction in PPR was also observed in the hippocampus of hypothyroid neonatal rats, and this effect resulted from increased neurotransmitter release during the first stimulus [27]. Our results suggest that like early hypothyroidism, a lack of thyroid hormones in the adult brain may also disrupt short-term synaptic plasticity.

When we examined the potential molecular mechanisms that could be responsible for the changes in electrophysiological parameters that we observed in the studied models of depression and hypothyroidism, we considered the participation of synaptotagmin I, the main Ca2+ sensor, postsynaptic proteins, metabolic disturbances, changes in mitochondrial dynamics, neurotrophic factors, alterations in the intensity of oxidative stress, intracellular signaling pathways and the level of acetylcholine, a neurotransmitter involved in cognitive processes. We demonstrated increased expression of hippocampal synaptotagmin I in WKY rats with and without PTU treatment. Synaptotagmin I, the primary Ca2+ sensor, is associated with action potential-triggered neurotransmitter release. An elevated level of synaptotagmin I in the hippocampus of hypothyroid neonatal rats was linked with enhanced glutamate release and a reduction in PPR [27]. The reduction in PPR and the upregulation of synaptotagmin I expression in the WKY rats suggests that this mechanism may also be altered in adult rats of this strain. In contrast to presynaptic neurotransmitter release, we did not observe any changes in the examined postsynaptic mechanisms, that is, the NMDA receptor subunits in the tested models.

The function of thyroid hormones in the periphery is mainly related to metabolism, but the present research reveals that these hormones, not only in the pre- and early postnatal periods, but also in the adult brain, affect metabolic processes and synaptic plasticity. In our previous studies, we found that in WKY rats, as well as in Wistar and WKY rats treated with PTU, glycolysis was reduced, and in a model of the coexistence of depression and hypothyroidism (WKY + PTU), the efficiency of mitochondrial respiration was impaired [12]. The increase in glycogen synthase expression observed in the present study, together with the lack of changes in glycogen phosphorylase in WKY animals with and without PTU treatment, suggests a shift in the balance towards glycogen synthesis and limiting the process of glycogenolysis. Many studies have indicated that glycogenolysis in astrocytes is an important source of lactate, which is delivered to neurons and, as a substrate for mitochondrial metabolism, plays a critical role in learning and memory mechanisms [28]. Thus, in WKY rats and in rats with a decrease in thyroid hormones, energy deficiencies may occur not only due to lower glycolysis (as in our previous study), but also as an effect of increased glycogen synthesis.

To obtain sufficient energy necessary for the function of neurons, proper mitochondrial dynamics are also important. The higher levels of dnm1l, a marker of mitochondrial fission, observed in animals of both strains receiving PTU suggest that a reduction in thyroid hormones disrupts the dynamics of mitochondria. However, the increased expression of opa1, a marker of fusion, in WKY rats administered PTU is difficult to explain. The observed changes in the studied markers of mitochondrial dynamics could, on the one hand, increase energy shortages (in the case of increased dnm1l expression), but on the other hand, could be adaptive mechanisms limiting energy deficits (in the case of increased opa 1 expression). However, in the case of opa 1, the available data indicate that only a long isoform of this protein promotes mitochondrial fusion, whereas a short isoform intensifies fission instead; thus, the observed increase in opa1 expression did not necessarily lead to increased fusion [29].

It is also known that oxidative damage in the brain may lead to cognitive impairments, and hypothyroidism-induced oxidative stress in the brain has been reported to contribute to learning and memory deficits. Additionally, a negative association between oxidative stress and BDNF levels has been shown [30]. However, in our experimental conditions, in both individual models and in the model of the co-occurrence of hypothyroidism and endogenous depression, this pathway does not seem to be directly or indirectly affected because we did not observe any changes in markers of oxidative stress (MDA and 4-HNE) or hippocampal BDNF and NGF expression, which suggests that learning and memory impairment does not occur through oxidative stress induction. Despite no changes in markers of oxidative stress, both in WKY rats, but mainly in the model of coexistence of depression and hypothyroidism, a strong increase of caspase-1 protein level was observed. Caspase-1 activation, mainly by NLRP3 inflammasome, may lead to the release of pro-inflammatory cytokines, and thus, intensify neuroinflammation. Current research also indicates the role of this enzyme in the regulation of neuronal plasticity. It has also been shown that caspase-1, by influencing the function of the AMPA receptor, weakens the LTP process in the hippocampus. However, in the models tested, the caspase-1 expression did not correlate with LTP values, suggesting that the disturbance in LTP was not due to activation of this enzyme. On the other hand, the increased level of this enzyme, especially in the model of coexistence of depression and hypothyroidism, may suggest the induction of the neuroinflammatory process.

In the WKY rats compared to the control Wistar rats, a reduction in LTP was observed in both the CA1 and DG hippocampal regions. LTP is known to be controlled at the molecular level by the activation of a number of neuronal signaling pathways, mainly the Ca-calmodulin-dependent kinase (CaMK), phosphatidylinositol-3 kinase/protein kinase B (PI3K/AKT), protein kinase A, protein kinase C, and mitogen-activated protein kinase (MAPK) pathways. The reduction in LTP observed in the WKY rats may be associated with a lower level of the active form of CaMKII (p-CaMKII) in the hippocampus, since CaMKII inhibition blocks LTP induction and phosphorylation of AMPA receptor subunits [31]. In addition to CaMKII, a lowered ratio of phospho-Akt/Akt was also observed in the WKY rats, so this kinase could also be responsible for LTP disturbance in this strain of rats. Given the potentiating effect of acetylcholine on LTP, the lower levels of this neurotransmitter in the hippocampus of the WKY rats could also potentially cause the weakening of the LTP in this strain. However, since hypothyroidism did not decrease the levels of p-CaMKII, p-Akt, or acetylcholine in either the Wistar or WKY rats in the present study, none of these factors seem to be responsible for the reduction in LTP in the dentate gyrus of the hippocampus observed in Wistar rats receiving PTU. Notably, an increase in the expression of p-ERK1-MAP kinase was shown in the hippocampus of rats in the group with coexisting depression and hypothyroidism (WKY + PTU). The fact that ERK activation was also observed after LTP stimulation in hippocampal slices from adult rats with hypothyroidism induced in the pre- and postnatal periods, as well as in the hippocampus of newborn rat pups born to hypothyroid dams, suggests that disturbances in synaptic plasticity associated with a deficiency of thyroid hormones result from changes in ERK signaling [11,32].

In summary, this study demonstrated that thyroid hormone deficiency in adult animals disturbed electrophysiological parameters of basal excitatory transmission, short-term synaptic plasticity, and long-term potentiation, as well as biochemical markers of neuronal plasticity in the hippocampus; these changes were similar to those observed with pre-or early postnatal hypothyroidism. Moreover, changes in the investigated markers of synaptic plasticity were also observed in WKY rats, a model of endogenous depression, either under baseline conditions or in response to a decrease in thyroid hormone levels. However, the limitation of our study is the measurement of only the gene expression of some factors. Future protein studies will probably provide additional answers. Although in the model of the co-occurrence of depression and hypothyroidism, stronger changes in PPR in the CA1 region of the hippocampus, the appearance of changes in the level of the active form of ERK, and an increase in the active form of caspase-1 were observed, the changes in basal excitatory transmission were not intensified and, in the case of long-term potentiation in the CA1 region were even decreased, probably due to the substantial differences between the Wistar and WKY strains. These data are currently difficult to interpret since the functional role of thyroid hormones in synaptic plasticity in adults, as well as the role of hypothyroidism in the pathogenesis of depression, has only begun to be elucidated.

## 4. Materials and Methods 

### 4.1. Animals and Treatments

All procedures were carried out to minimize animal suffering and reduce the number of animals used (3R policy). The study protocol was approved by the Local Ethics Committee, Kraków, Poland (Permit No. 46/2018 of 01.02.2018).

Male Wistar and Wistar–Kyoto rats (aged 8 weeks upon arrival) were obtained from Charles-River Laboratories (Charles River Laboratories, Hamburg, Germany) and were maintained under standard conditions (room temperature of 23 °C, 12/12-h light/dark cycle, lights on at 6:00 a.m., and ad libitum access to water and food). After quarantine, the rats were randomly assigned into the following four groups: Group I (Control): Wistar rats fed a standard diet and drinking water ad libitum; Group II (Endogenous depression): Wistar–Kyoto rats fed a standard diet and drinking water ad libitum; Group III (Hypothyroid): Wistar rats fed a standard diet and treated with 0.05% (*w*/*v*) PTU in drinking water for three weeks; and Group IV (Coexistent depression and hypothyroidism): Wistar–Kyoto rats fed a standard diet and treated with 0.05% (*w*/*v*) PTU in drinking water for three weeks. PTU prevents thyroid hormone synthesis by inhibiting thyroid peroxidase-catalyzed reactions, blocking iodine organization and coupling of iodotyrosines, and inhibiting the peripheral deiodination of T4 to T3. After 3 weeks of treatment, the animals underwent behavioral tests.

### 4.2. Behavioral Testing

#### 4.2.1. Elevated Plus Maze (EPM) Test

The elevated plus maze (EPM) is a test that measures anxiety behavior in laboratory animals. The EPM, which consisted of two open and two closed arms, was 50 cm above the floor and illuminated from beneath with only a dim light (15 W). To allow the animals to adapt to the experimental conditions, they were placed in the experimental room for one hour before the test. During the 5-min test, each rat was placed in the center of the EPM facing the open arms, and the following parameters were measured: The time spent in and the number of entries into the open and closed arms [33]. The percentage of open arm entries ([open entries]/[total entries] × 100) and the percentage of time spent in the open arms ([time spent in the open entries]/[time spent in both arms] × 100) were calculated.

#### 4.2.2. Novel Object Location (NOL) Test

The NOL test was used to determine spatial working memory. This test is based on the spontaneous tendency of rats to spend more time exploring an object in a new location. On the first day, the rats were put into the empty container for habituation to the environment. The following day, two identical objects were added into the container in opposite corners 15 cm from the walls. The rats spent 3 min exploring the objects (NOL training phase). After a 1 h retention interval, one object was shifted by 20 cm, and the rats were allowed to explore the objects for another 3 min (NOL test phase). During the test, the time that animals spent exploring both objects was measured. The discrimination index (DI) was calculated based on results of the NOL test phase by the following equation: DI = ([time with novel location − time with familiar location])/[time with novel location + time with familiar location]) [34].

### 4.3. Electrophysiological Study

#### 4.3.1. Slice Preparation

The animals were anesthetized with isoflurane (Aerrane, Baxter, Deerfield, IL, USA) and decapitated under nonstressed conditions as described previously [35]. The isolated brains were immersed in ice-cold artificial cerebrospinal fluid (aCSF) containing (in mM) 130 NaCl, 5 KCl, 2.5 CaCl_2_, 1.3 MgSO_4_, 1.25 KH_2_PO_4_, 26 NaHCO_3_, and 10 D-glucose (Sigma-Aldrich, Saint Louis, MO, USA). During the whole preparation procedure, aCSF was bubbled with a mixture of 95% O_2_ and 5% CO_2_. The hippocampi were dissected and cut into transverse slices (400 µm thick) using a vibrating microtome (Leica VT1000S, Leica Biosystems, Wetzlar, Germany).

#### 4.3.2. Field Potential (FP) Recording and LTP Induction

The slices were placed in the recording interface chamber and superfused (2.5 mL/min) with warm (32 ± 0.5 °C), modified aCSF composed of (in mM): 132 NaCl, 2 KCl, 2.5 CaCl_2_, 1.3 MgSO_4_, 1.25 KH_2_PO_4_, 26 NaHCO_3_, and 10 D-glucose bubbled with 95% O_2_ and 5% CO_2_. A bipolar stimulating electrode (FHC, Bowdoin, ME, USA) was placed in the Schaffer collateral pathway or perforant path in the hippocampal slices. Stimuli with a duration of 0.2 ms were applied at 0.033 Hz using a constant-current stimulus isolation unit (WPI, Friedberg, Germany). Glass micropipettes filled with aCSF (1–3 MΩ) were used to record field potentials. The recording electrode was placed within the stratum radiatum of the CA1 area or in the dentate gyrus (DG) in the hippocampal slices. Recorded responses were amplified (EXT 10-2F amplifier, NPI, Tamm, Germany), filtered (1 Hz–1 kHz), A/D converted (10 kHz sampling rate), and stored on a PC using the Micro1401 interface and Signal 4 software (CED, Cambridge, UK).

A stimulus-response (input-output) curve was made for each brain slice. Afterward, stimulation intensity was adjusted to evoke a response of 30% of the maximum amplitude. For the induction of LTP in the hippocampus, a protocol of high-frequency stimulation (HFS) was used. The HFS consisted of three trains of 100 pulses at 100 Hz. Each train lasted 1 s, and the gap between them was 3 min.

The stimulus-response curves obtained for each slice were fit with the Boltzmann equation: Vi = Vmax/(1 + exp ((u − uh)/− S)). The following parameters were compared: The maximum field potential amplitude (Vmax), the stimulation intensity evoking a field potential of half-maximum amplitude (uh), and the factor proportional to the slope of the curve (S). The first 15 min of recording was the baseline (100%) against which subsequent measurements were normalized. The amount of LTP was determined as an average increase in the slope of the descending phase of FPs recorded between 60–75 min after stimulation relative to baseline.

### 4.4. Biochemical Analysis

The rats were sacrificed under nonstressed conditions by rapid decapitation. The brains were rapidly removed, and the hippocampi were dissected on ice-cold glass plates. Tissues were frozen on dry ice and stored at −80 °C until use in further biochemical analysis.

#### 4.4.1. Gene Expression Measurement: RNA Extraction, cDNA Preparation and Real-Time PCR

The procedure was performed as described previously [12]. Briefly, total RNA was extracted using a Total RNA Mini Kit (A&A Biotechnology, Gdynia, Poland) following the manufacturer’s instructions. RNA concentration was measured using a NanoDrop ND-1000 spectrometer (Thermo Fisher Scientific, Waltham, MA, USA). RNA was reverse transcribed into cDNA using a commercial kit for reverse transcription (Thermo Fisher Scientific, Waltham, MA, USA) according to the manufacturer’s instructions.

TaqMan gene expression assays for the following genes were used: syt1, opa1, gys1, dnm1l, bdnf, ngf, and pygb (all Thermo Fisher Scientific, Waltham, MA, USA). Amplification was performed using a 20 μL mixture containing PCR Master Mix, reverse-transcribed cDNA, TaqMan forward and reverse primers, and a hydrolysis probe (250 nM) labeled at the 5′-end with the fluorescent reporter FAM and at the 3′-end with a quenching dye. The thermal cycling conditions were 2 min at 50 °C and 10 min at 95 °C followed by 40 cycles at 95 °C for 15 s and 60 °C for 1 min. The samples were run in a CFX96 Real-Time System (Bio-Rad, Hercules, CA, USA). hprt1 (Thermo Fisher Scientific, Waltham, MA, USA) was used as the housekeeping gene. The ∆∆Ct method was used for data analysis, and the results were calculated as the fold change in expression compared to that in the control Wistar strain.

#### 4.4.2. Tissue Preparation and Determination of Protein Concentration

Tissue samples were homogenized in 2-mL tubes filled with an appropriate buffer using a Tissue Lyser II (Qiagen Inc., Valencia, CA, USA). The protein level was measured with the BCA method [36] using a BCA Protein Assay Kit (Sigma-Aldrich, Saint Louis, MO, USA) according to the supplier’s instructions using a spectrophotometer (Tecan Infinite M200 Pro, Männedorf, Switzerland) at a wavelength of 562 nm. The sample extracts were stored at −80 °C until use in enzyme-linked immunosorbent assays (ELISAs) or Western blot analysis.

#### 4.4.3. ELISA

The levels of acetylcholine (Biovision, Milpitas, CA, USA), malondialdehyde (MDA), 4-hydroxynonenal (4-HNE) (both from CellBiolabs, San Diego, CA, USA), AKT1, and p-AKT1 (S473) (both from Bioassay Technology Laboratory, Shanghai, China) in the hippocampal homogenates were measured using commercially available ELISA kits according to the instructions provided by the manufacturers. For each ELISA, the samples were prepared in accordance with the supplier’s recommendations. The absorbance was measured using a Tecan Infinite M200 Pro (TECAN, Männedorf, Switzerland) set to the appropriate wavelength. The intra- and interassay precision was dependent on the properties of the assay.

#### 4.4.4. Western Blot

Western blotting was performed to detect NMDA receptor subunits (GluN1, GluN2A, GluN2B), Calcium/calmodulin-dependent protein kinase II isoform K (total and phospho-CaMKII form), CREB (total and phospho-(Ser133) form), Extracellular signal-regulated kinases 1 and 2 (total and phospho-ERK 1/2 form) and caspase-1.

Hippocampal samples homogenized in 2% SDS (BioShop, Burlington, ON, Canada) containing equal amounts of protein were mixed with gel loading buffer (Bio-Rad, CA, Hercules, CA, USA) and boiled at 95 °C for 5 min. The proteins were separated using Criterion™ TGX™ Precast Midi Protein Gels (Bio-Rad, Hercules, CA, USA) under constant voltage (150 V) and then transferred to PVDF membranes (Sigma-Aldrich, Saint Louis, MO, USA). After semidry transfer, some membranes were cut to allow simultaneous incubation with different antibodies. The membranes were blocked in 5% skim milk in Tris-buffered saline (TBS) with 0.05% Tween 20 (Sigma-Aldrich, Saint Louis, MO, USA) for 1 h at room temperature (RT) and incubated overnight at 4 °C with primary antibodies against the following proteins GluN1, GluN2A, GluN2B (Santa Cruz Biotechnology, Dallas, TX, USA), phospho-CaMKII (Abcam, Cambridge, UK), phospho-CREB (Ser133) (Merck Millipore, Burlington, MA, USA), phospho-ERK1/ERK2 (R&D Systems, Minneapolis, MN, USA), caspase-1 (Santa Cruz Biotechnology, Dallas, TX, USA). All antibodies were diluted using a SignalBoost Immunoreaction Enhancer Kit (Merck, Darmstadt, Germany). The next day, the membranes were washed 4 times for 10 min each in TBST (TBS with 0.05% Tween 20) and incubated at RT with horse anti-mouse/goat anti-rabbit/mouse anti-goat IgG HRP peroxidase-conjugated secondary antibody (Vector Laboratories, Peterborough, UK; Santa Cruz Biotechnology, Dallas, TX, USA) for 1 h. After washing 4 times for 10 min each in TBST (Tris-Buffered saline with 0.05% Tween 20), the bands were detected using BM Chemiluminescence Western Blotting Substrate (POD) (Roche, Mannheim, Germany). Chemiluminescence was recorded using a luminescent image analyzer with a Fujifilm LAS-1000 System (Fujifilm, Tokyo, Japan). The relative levels of immunoreactivity were densitometrically quantified using Fujifilm Multi Gauge software (Fujifilm, Tokyo, Japan). The membranes were stripped twice using stripping buffer containing 100 mL of Tris-HCl (pH = 6.7), 2% SDS, and 700 μL of 2-mercaptoethanol (all from Sigma-Aldrich, Saint Louis, MO, USA); washed 3 times for 10 min each in TBST; blocked; and reprobed with antibodies against total forms of the measured proteins (CaMKII: Abcam, Cambridge, UK; CREB: Merck Millipore, Burlington, MA, USA; ERK1/ERK2: R&D Systems, Minneapolis, MN, USA) or with an antibody against β-actin (Sigma-Aldrich, Saint Louis, MO, USA). In the case of caspase-1 vinculin (Sigma-Aldrich, Saint Louis, MO, USA) was used as an internal loading control. The membranes were included in the Supplementary Materials.

#### 4.4.5. Statistics

All graphs were prepared using GraphPad Prism 9 (San Diego, CA, USA). The results of the experiments are presented as the means ± SEM. Statistical analysis of behavioral and biochemical data was performed using Statistica 13.3 software (StatSoft, Palo Alto, CA, USA) and consisted of two-way analyses of variance (ANOVAs) followed by the Duncan post hoc test when appropriate. Differences were considered significant at *p* < 0.05. Statistical evaluation of electrophysiological data was performed with GraphPad Prism 9 (San Diego, CA, USA), two-way analyses of variance (ANOVAs), and post hoc Tukey tests. The ANOVA results are reported as an F-statistic and its associated degrees of freedom.

## Figures and Tables

**Table 1:** Input-output relationship plots in the CA1 region of the hippocampus. The results are expressed as the mean ± SEM. * *p* < 0.05 vs. the control group (Wistar rats); ^^^ *p* < 0.001 vs. Wistar PTU group. WKY, Wistar–Kyoto.

Group	Amplitude V_max_ (mV)	U_50_ (µA)	Slope	Number of Slices
Wistar	2.50 ± 0.17	18.54 ± 1.10	7.02 ± 0.55	26
WKY	2.42 ± 0.13	16.54 ± 1.30	7.03 ± 0.66	24
Wistar + PTU	3.15 ± 0.17 *	21.79 ± 1.20	7.05 ± 0.50	22
WKY + PTU	1.95 ± 0.09 ^^^	17.45 ± 1.02	7.05 ± 0.61	18

**Table 2:** Input-output relationship plots in the dentate gyrus (DG) region of the hippocampus. The results are expressed as the mean ± SEM. * *p* < 0.05 vs. the control group (Wistar rats); ^^^ *p* < 0.001 vs. Wistar rats treated with PTU.

Group	Amplitude V_max_ (mV)	U_50_ (µA)	Slope	Number of Slices
Wistar	2.54 ± 0.13	13.51 ± 0.96	3.96 ± 0.53	15
WKY	1.90 ± 0.15 *	15.01 ± 1.00	5.42 ± 0.51	20
Wistar + PTU	3.18 ± 0.16 *	14.71 ± 0.65	4.69 ± 0.37	24
WKY + PTU	2.02 ± 0.14 ^^^	13.14 ± 0.78	5.12 ± 0.59	16

**Table 3:** Baseline-corrected paired-pulse ratio (PPR) in the CA1 and DG regions of the hippocampus. The results are expressed as the mean ± SEM. ** *p* < 0.01, *** *p* < 0.001 vs. the control group (Wistar rats) ## *p*< 0.01 vs. WKY group; ^ *p* < 0.05, ^^ *p* < 0.01 vs. Wistar PTU group.

Group	CA1	DG
Wistar	1.478 ± 0.024	0.8007 ± 0.011
WKY	1.359 ± 0.029 **	0.7955 ± 0.011
Wistar + PTU	1.347 ± 0.022 **	0.7898 ± 0.012
WKY + PTU	1.239 ± 0.019 *** ## ^	0.7304 ± 0.01 *** ## ^^

**Table 4:** Mean slope of FPs recorded between 45 and 60 min after HFS in the CA1 and DG regions of the hippocampus. * *p* < 0.05, ** *p* < 0.01, *** *p* < 0.001 vs. the control group (Wistar rats); ### *p* < 0.001 vs. WKY group.

Group	CA1—fEPSP Slope	DG—fEPSP Slope
Wistar	172.7 ± 1.93	162.5 ± 0.965
WKY	145.4 ± 1.91 ***	137.1 ± 0.87 **
Wistar + PTU	181.2 ± 2.16 *	127.1 ± 1.46 ***
WKY + PTU	175.3 ± 1.62 ###	140.7 ± 1.018 *

**Table 5:** The effects of strain and PTU treatment on the protein levels of subunits of NMDA receptor (GluN1, GluN2A, GluN2B) in the hippocampus. The results are expressed as the average fold change ± SEM. *n* = 7–10.

Group	GluN1	GluN2A	GluN2B
Wistar	1.00 ± 0.09	1.00 ± 0.07	1.00 ± 0.05
WKY	1.02 ± 0.07	1.03 ± 0.06	0.87 ± 0.05
Wistar + PTU	0.91 ± 0.09	0. 99 ± 0.08	0.93 ± 0.09
WKY + PTU	1.02 ± 0.11	1.07 ± 0.08	0.86 ± 0.06
	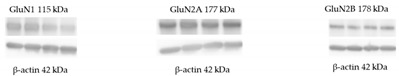		

## Data Availability

The data that support the findings of this study are available from the corresponding author upon reasonable request from qualified researchers.

## Supplementary Materials

The following are available online at https://www.mdpi.com/1422-0067/22/4/1599/s1.

## Abbreviations

ANOVA	analysis of variance
BDNF	brain-derived neurotrophic factor
CaMK II	calcium/calmodulin-dependent protein kinase II
CAPS	3-clohexylaminopropanesulfonic acid
CREB	cAMP Response Element-Binding Protein
DG	dentate gyrus
Dnm1l	dynamin-1-like
ELISA	enzyme-linked immunosorbent assay
EPM	elevated plus maze
ERK	extracellular signal-regulated kinases
FP	field potential
Gys1	glycogen sythase
HPT	Hypothalamic—pituitary—thyroid
HFS	high frequency stimulation
LTP	long-term potentiation
MDA	malondialdehyde
NGF	nerve growth factor
NMDA	*N*-methyl-d-aspartate receptor
NOL	novel object location
PCR	polymerase chain reaction
Pygb	glycogen phosphorylase
PPR	paired-pulse ratio
PTU	propylthiouracil
PVDF	polyvinylidene fluoride
SDS-PAGE	SDS poliacrylamide gel electrophoresis
Syt1	synaptotagmin 1
T3	triiodothyronine
T4	thyroxine
TBS	Tris-buffered saline
TMB	3,3′,5,5′-tetramethylbenzidine
THs	thyroid hormones
TRE	thyroid response element
WKY	Wistar—Kyoto rats
4-HNE	4-hydroxynonenal
