# Supplementary material for: Contribution of Hypothyroidism to Cognitive Impairment and Hippocampal Synaptic Plasticity Regulation in an Animal Model of Depression
Source: Int J Mol Sci. 2021 Feb 5;22(4):1599. doi: 10.3390/ijms22041599 (PMC7915890; doi:10.3390/ijms22041599)
Supplement: Supplementary file 1 [file ijms-22-01599-s001.pdf]

GluN1

The membranes were cut after transfer at 75kDa to allow for simultaneous incubation with the antibody against β-actin

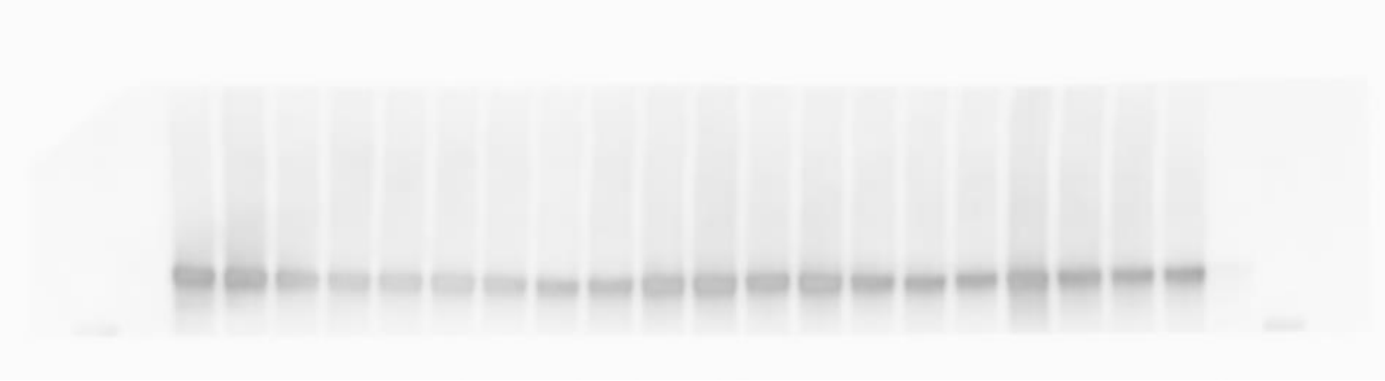

The bands from the left: Wistar, WKY, Wistar PTU, WKY PTU, Wistar, WKY, Wistar PTU, WKY PTU, Wistar, WKY, Wistar PTU, WKY PTU, Wistar, WKY, Wistar PTU, WKY PTU, Wistar, WKY, Wistar PTU, WKY PTU

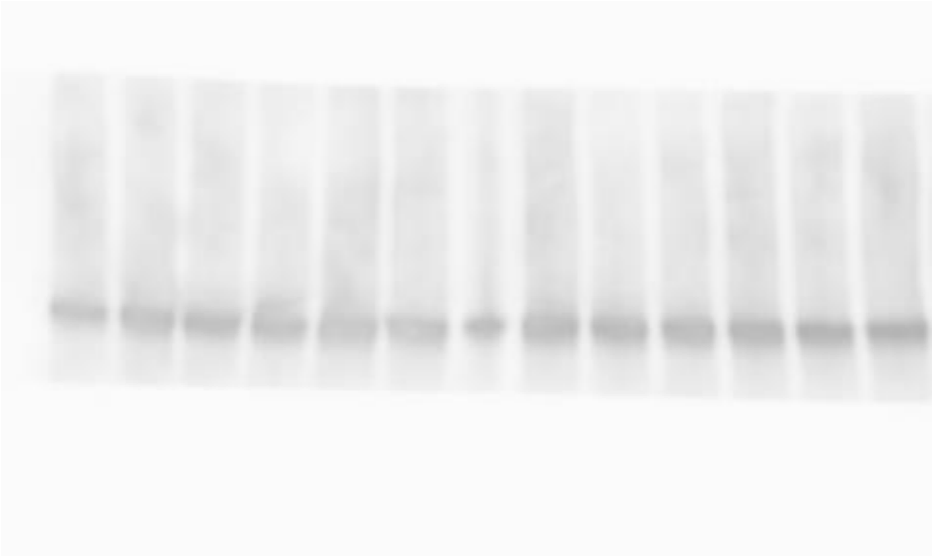

The bands from the left: Wistar, WKY, Wistar PTU, WKY PTU, Wistar, WKY, Wistar PTU, WKY PTU, Wistar, WKY, Wistar PTU, WKY PTU, Wistar

β-actin (as loading control) to GluN1

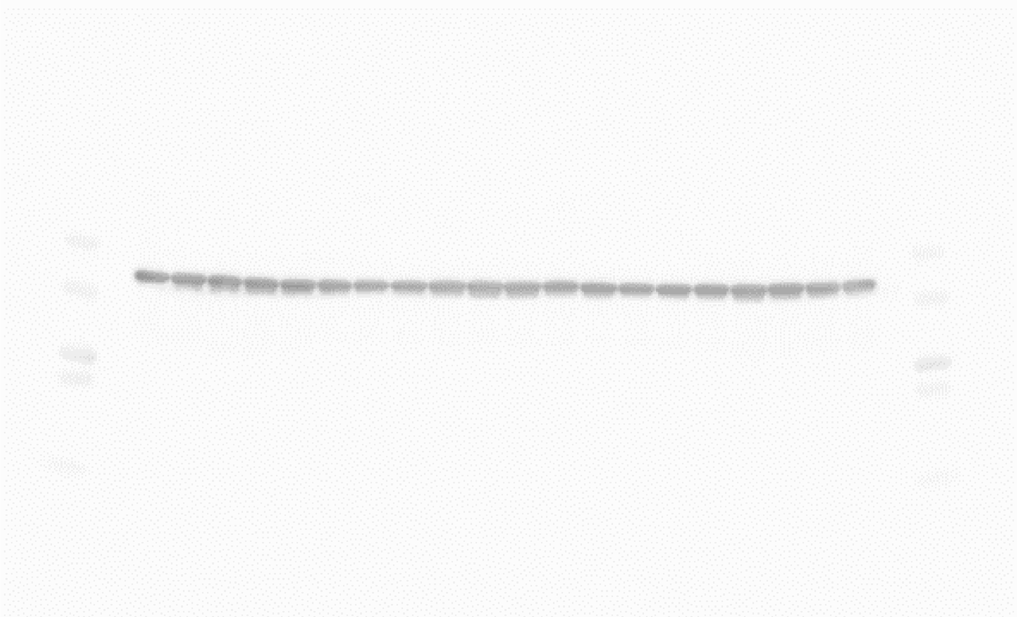

The bands from the left: Wistar, WKY, Wistar PTU, WKY PTU, Wistar, WKY, Wistar PTU, WKY PTU, Wistar, WKY, Wistar PTU, WKY PTU, Wistar, WKY, Wistar PTU, WKY PTU, Wistar, WKY, Wistar PTU, WKY PTU

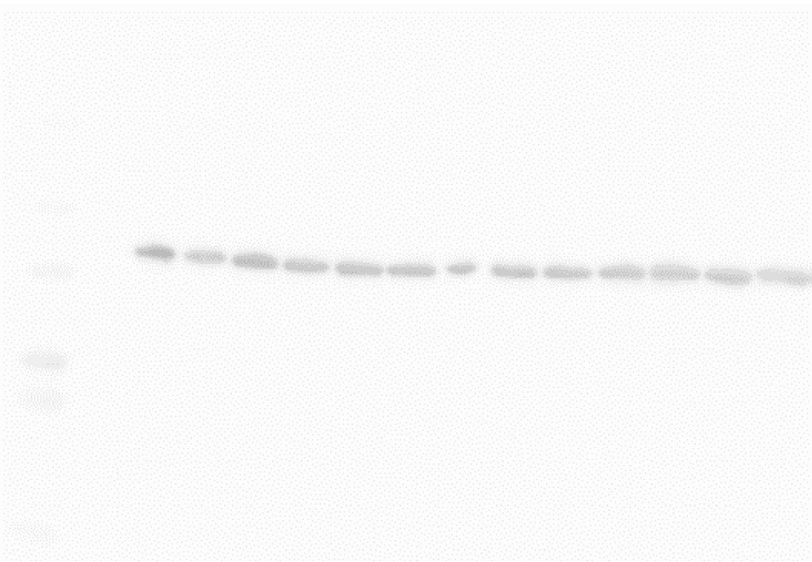

The bands from the left: Wistar, WKY, Wistar PTU, WKY PTU, Wistar, WKY, Wistar PTU, WKY PTU, Wistar, WKY, Wistar PTU, WKY PTU, Wistar

GluN2A

The membranes were cut after transfer at 75kDa to allow for simultaneous incubation with the antibody against β-actin

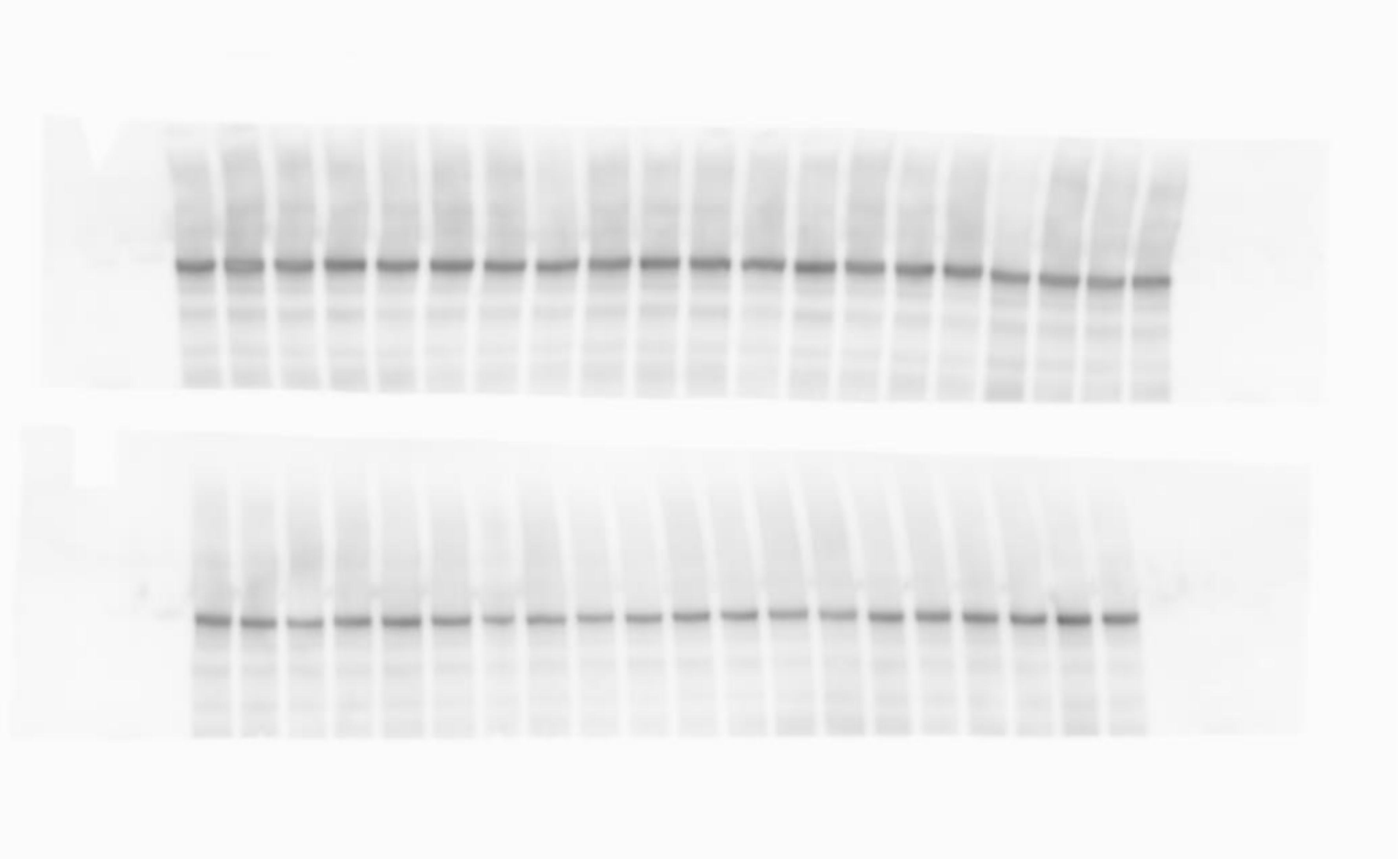

The bands from the left: Wistar, WKY, Wistar PTU, WKY PTU, Wistar, WKY, Wistar PTU, WKY PTU, Wistar, WKY, Wistar PTU, WKY PTU, Wistar, WKY, Wistar PTU, WKY PTU, Wistar, WKY, Wistar PTU, WKY PTU

β-actin (as loading control) to GluN2A

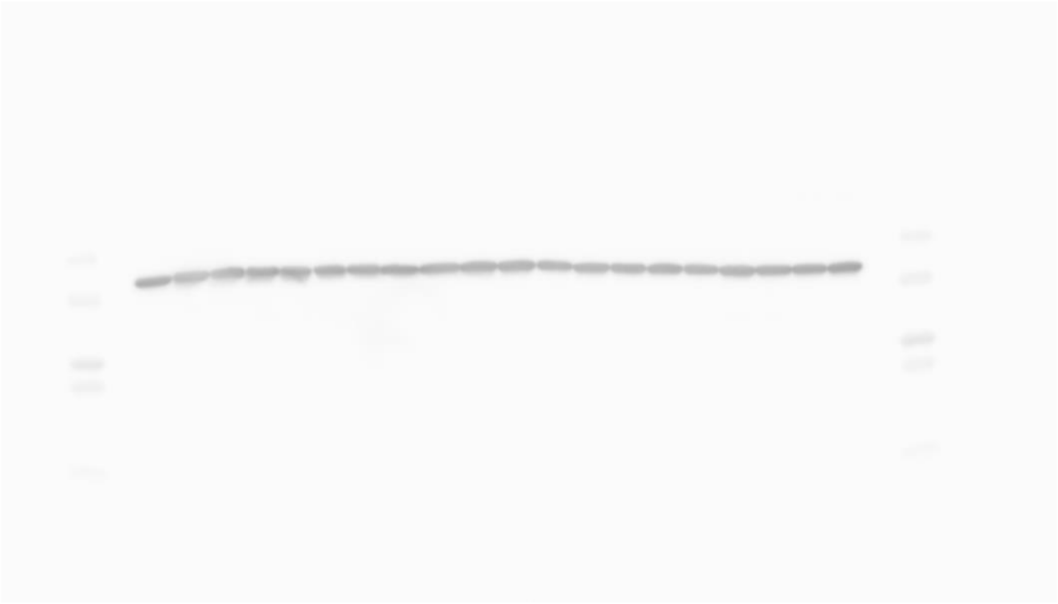

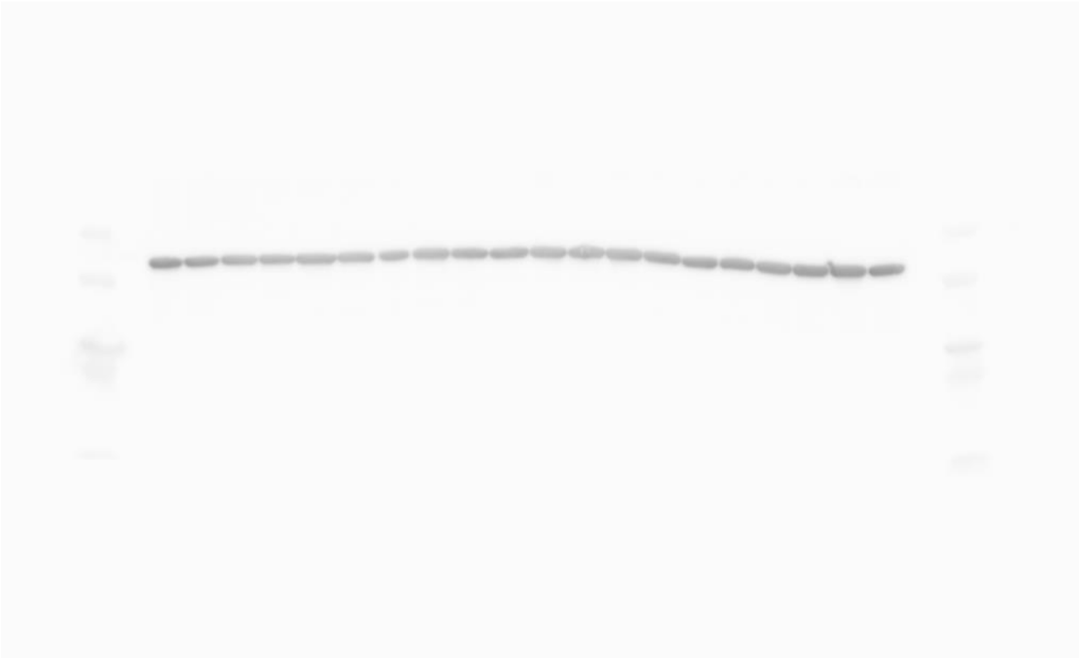

The bands from the left: Wistar, WKY, Wistar PTU, WKY PTU, Wistar, WKY, Wistar PTU, WKY PTU, Wistar, WKY, Wistar PTU, WKY PTU, Wistar, WKY, Wistar PTU, WKY PTU, Wistar, WKY, Wistar PTU, WKY PTU

GluN2B

The membranes were cut after transfer at 75kDa to allow for simultaneous incubation with the antibody against β-actin

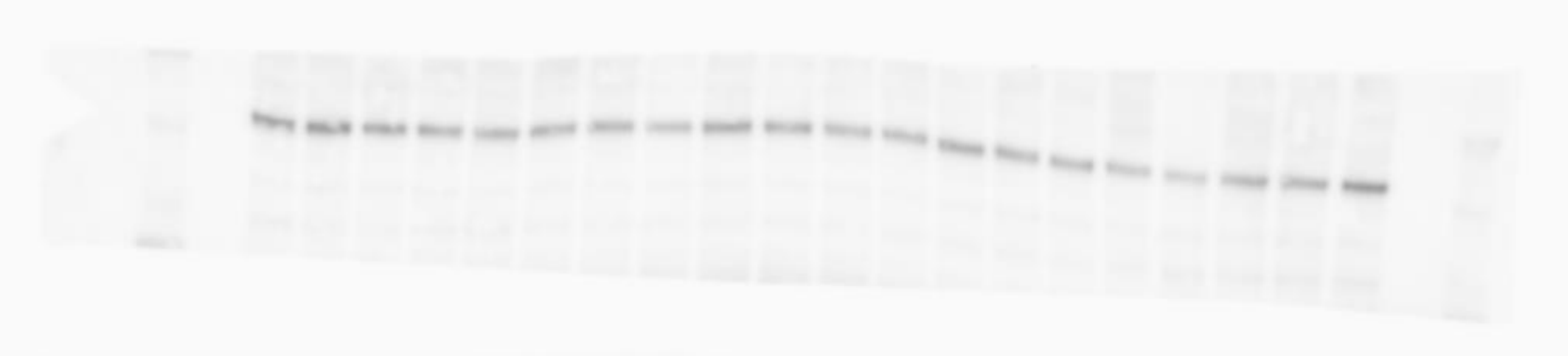

The bands from the left: Wistar, WKY, Wistar PTU, WKY PTU, Wistar, WKY, Wistar PTU, WKY PTU, Wistar, WKY, Wistar PTU, WKY PTU, Wistar, WKY, Wistar PTU, WKY PTU, Wistar, WKY, Wistar PTU, WKY PTU

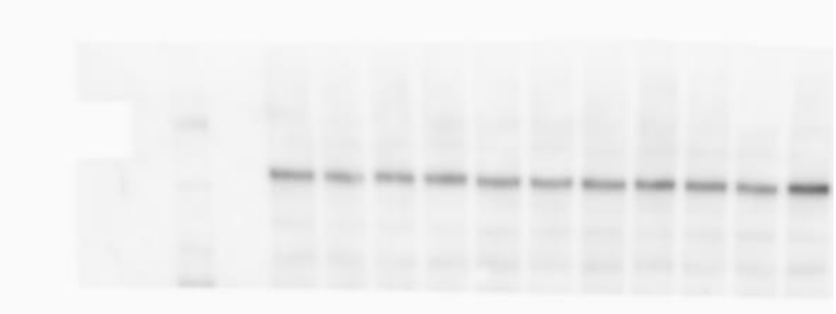

The bands from the left: Wistar, WKY, Wistar PTU, WKY PTU, Wistar, WKY, Wistar PTU, WKY PTU, Wistar, WKY, Wistar PTU

β-actin (as loading control) to GluN2B

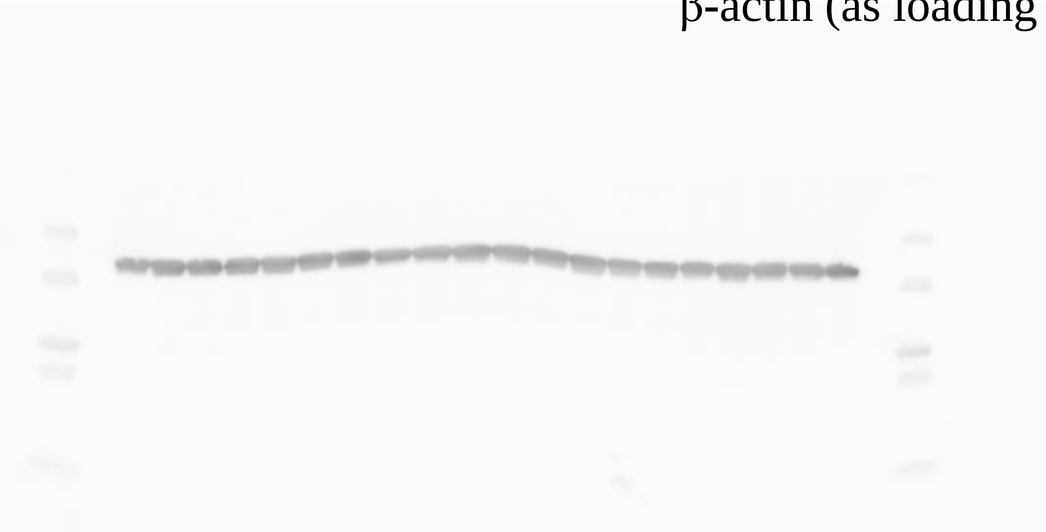

The bands from the left: Wistar, WKY, Wistar PTU, WKY PTU, Wistar, WKY, Wistar PTU, WKY PTU, Wistar, WKY, Wistar PTU, WKY PTU, Wistar, WKY, Wistar PTU, WKY PTU, Wistar, WKY, Wistar PTU, WKY PTU

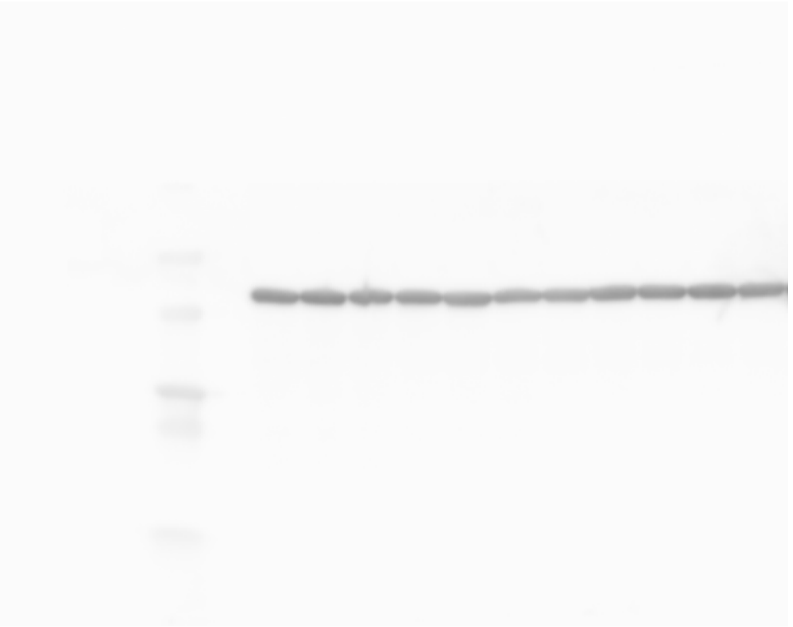

The bands from the left: Wistar, WKY, Wistar PTU, WKY PTU, Wistar, WKY, Wistar PTU, WKY PTU, Wistar, WKY, Wistar PTU

caspase-1

The membranes were cut after transfer to allow for simultaneous incubation with the antibody against Vinculin (loading conntrol)

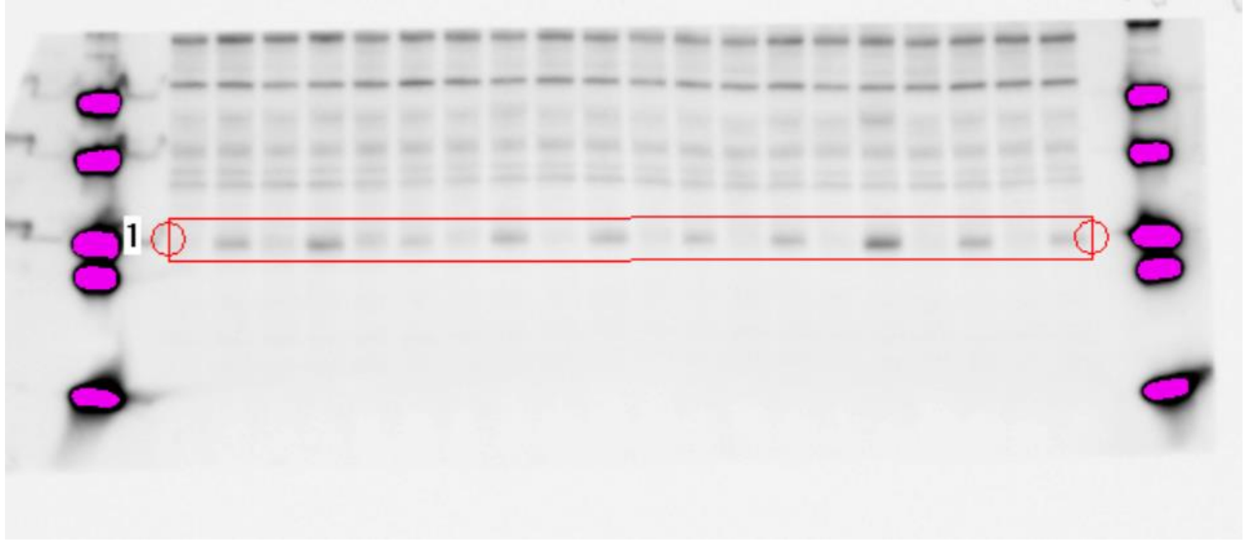

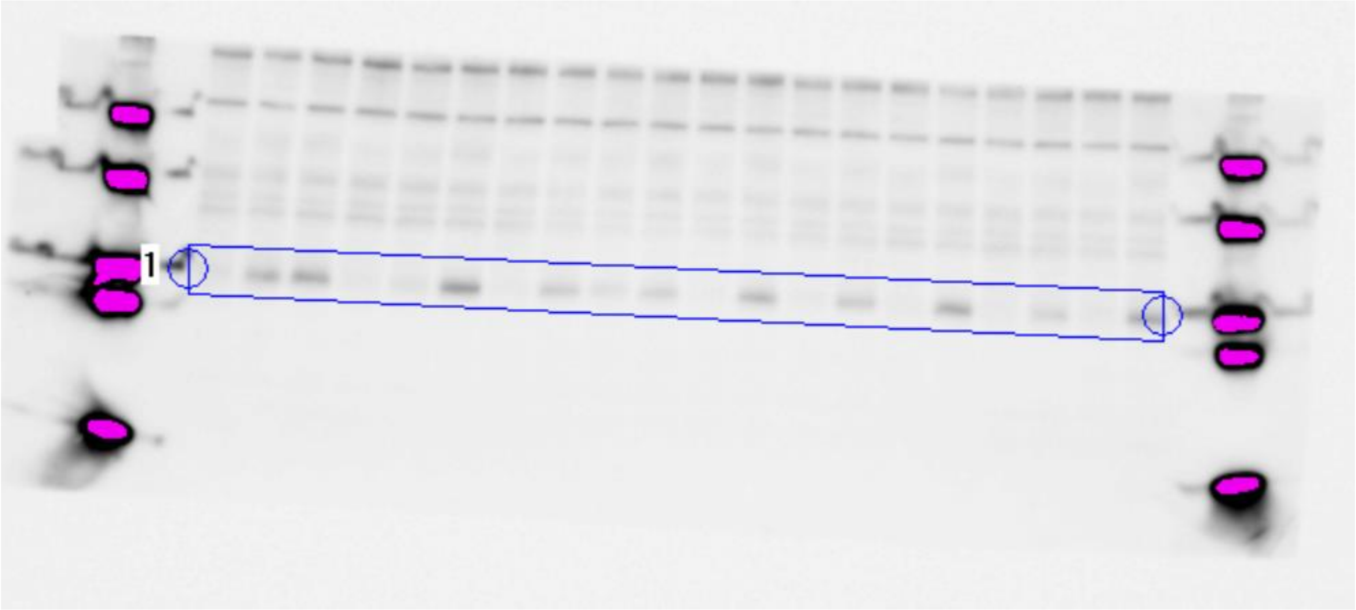

Vinculin (as loading control) to caspase-1

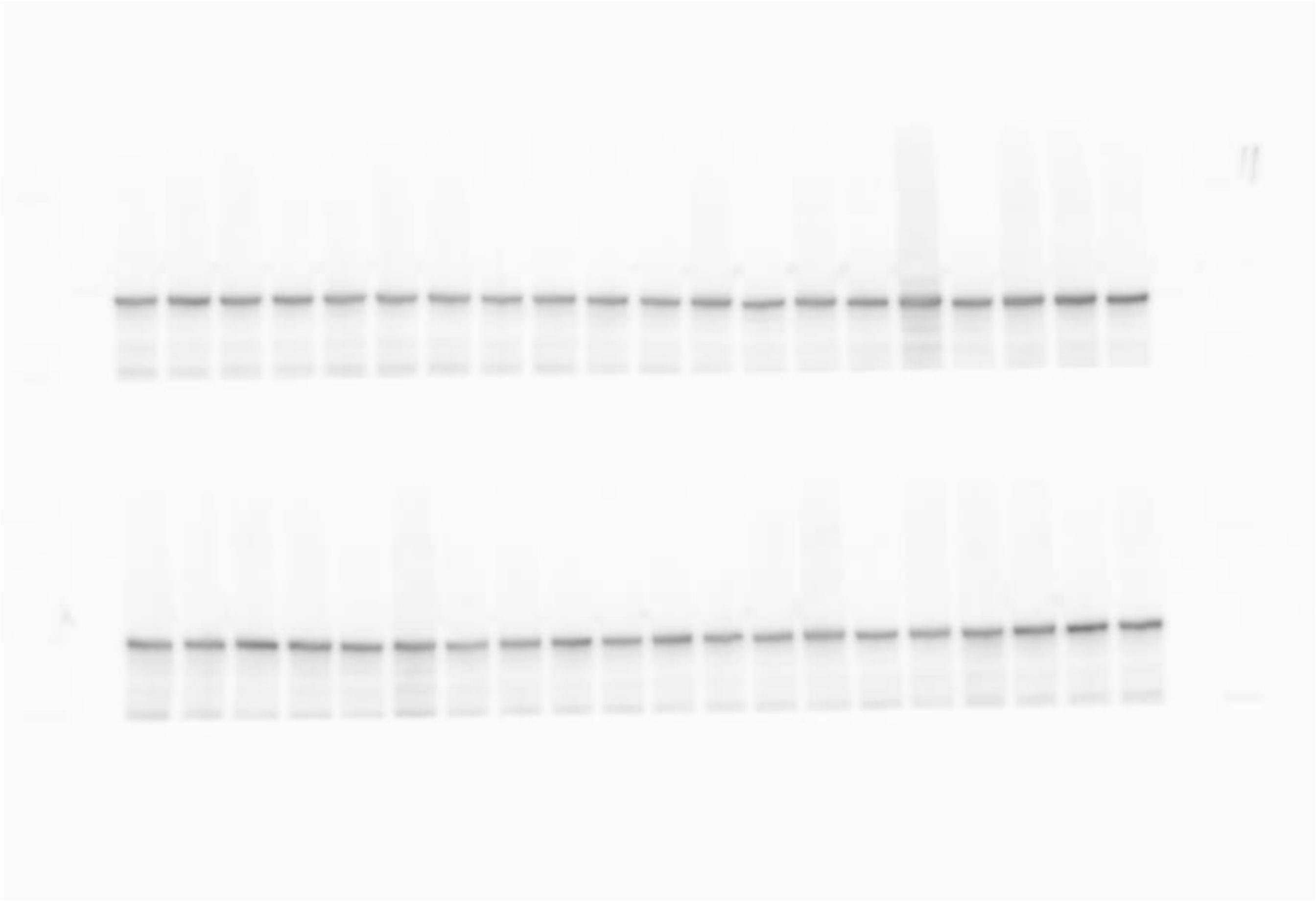

The bands from the left: Wistar, WKY, Wistar PTU, WKY PTU, Wistar, WKY, Wistar PTU, WKY PTU, Wistar, WKY, Wistar PTU, WKY PTU, Wistar, WKY, Wistar PTU, WKY PTU, Wistar, WKY, Wistar PTU, WKY PTU

p-CaMKII

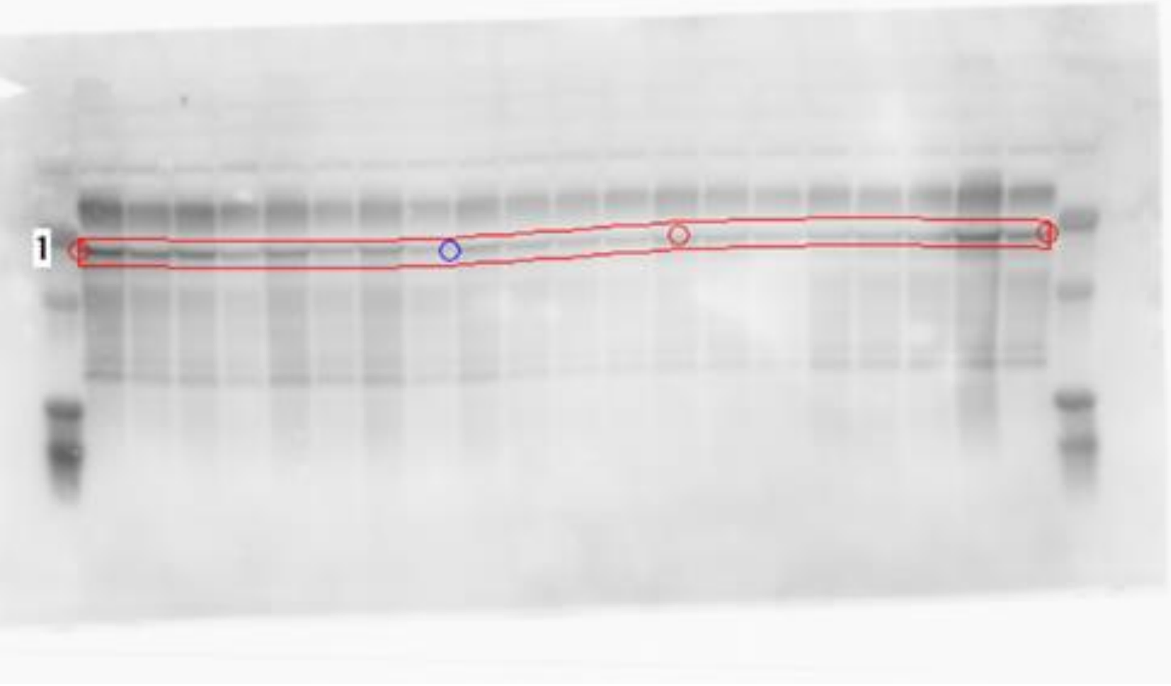

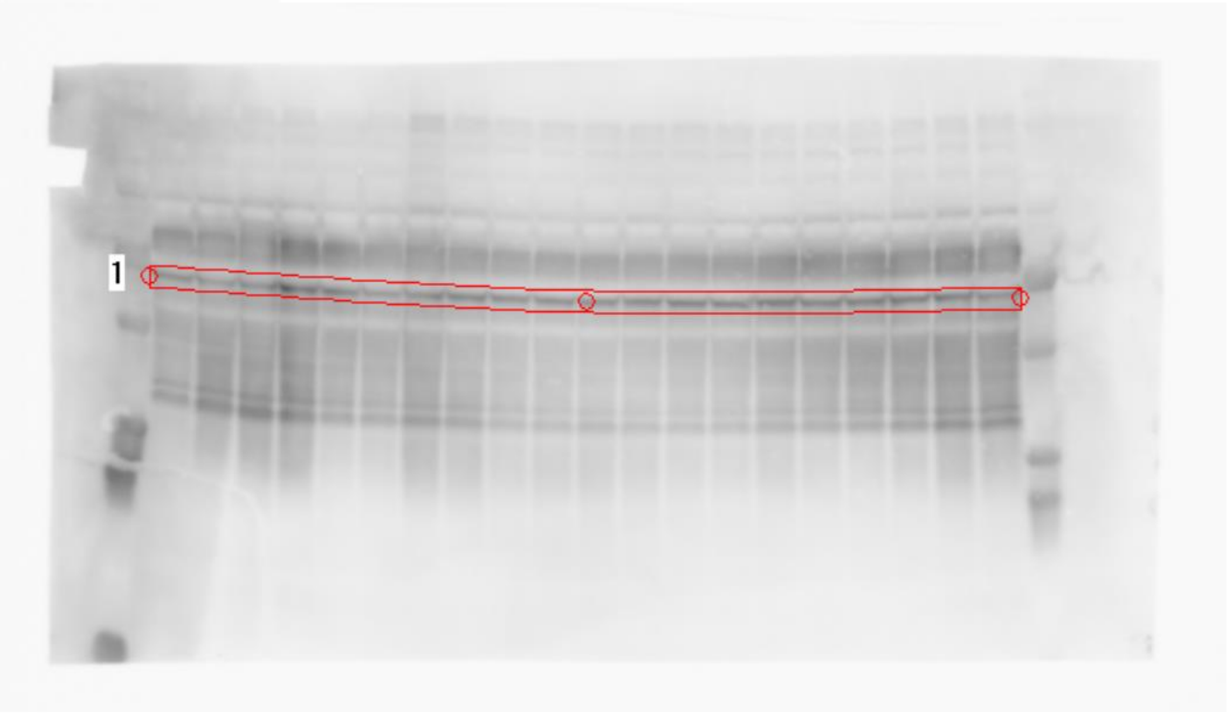

The bands from the left: Wistar, WKY, Wistar PTU, WKY PTU, Wistar, WKY, Wistar PTU, WKY PTU, Wistar, WKY, Wistar PTU, WKY PTU, Wistar, WKY, Wistar PTU, WKY PTU, Wistar, WKY, Wistar PTU, WKY PTU

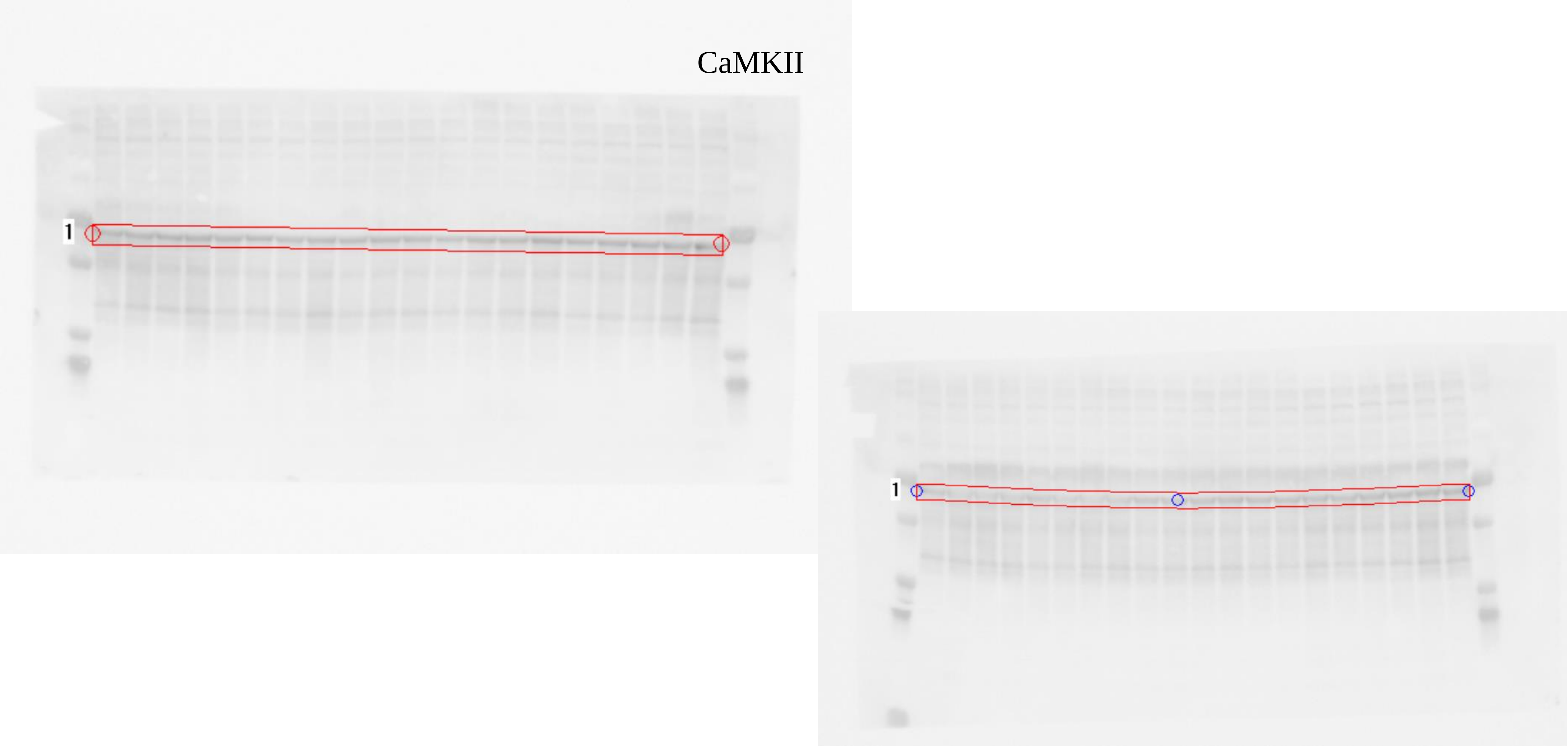

The bands from the left: Wistar, WKY, Wistar PTU, WKY PTU, Wistar, WKY, Wistar PTU, WKY PTU, Wistar, WKY, Wistar PTU, WKY PTU, Wistar, WKY, Wistar PTU, WKY PTU, Wistar, WKY, Wistar PTU, WKY PTU

β-actin (as loading control) to p-CaMKII and CaMKII proteins

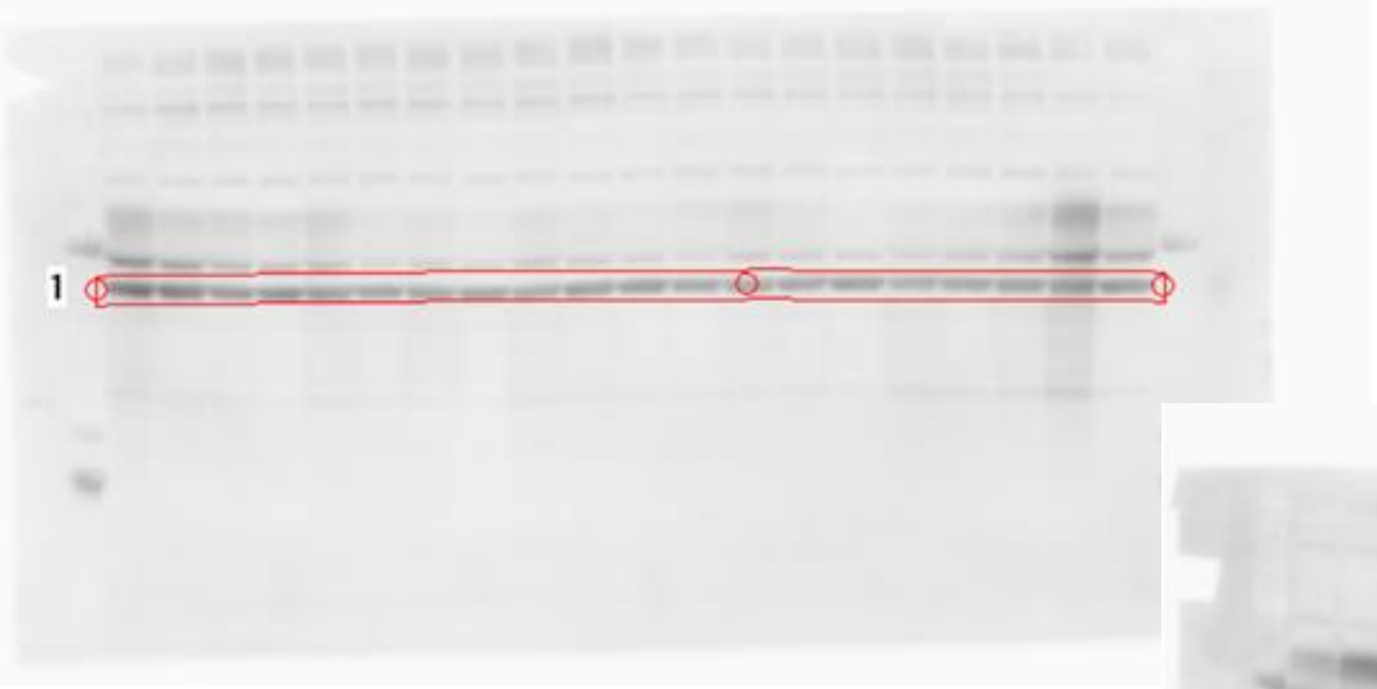

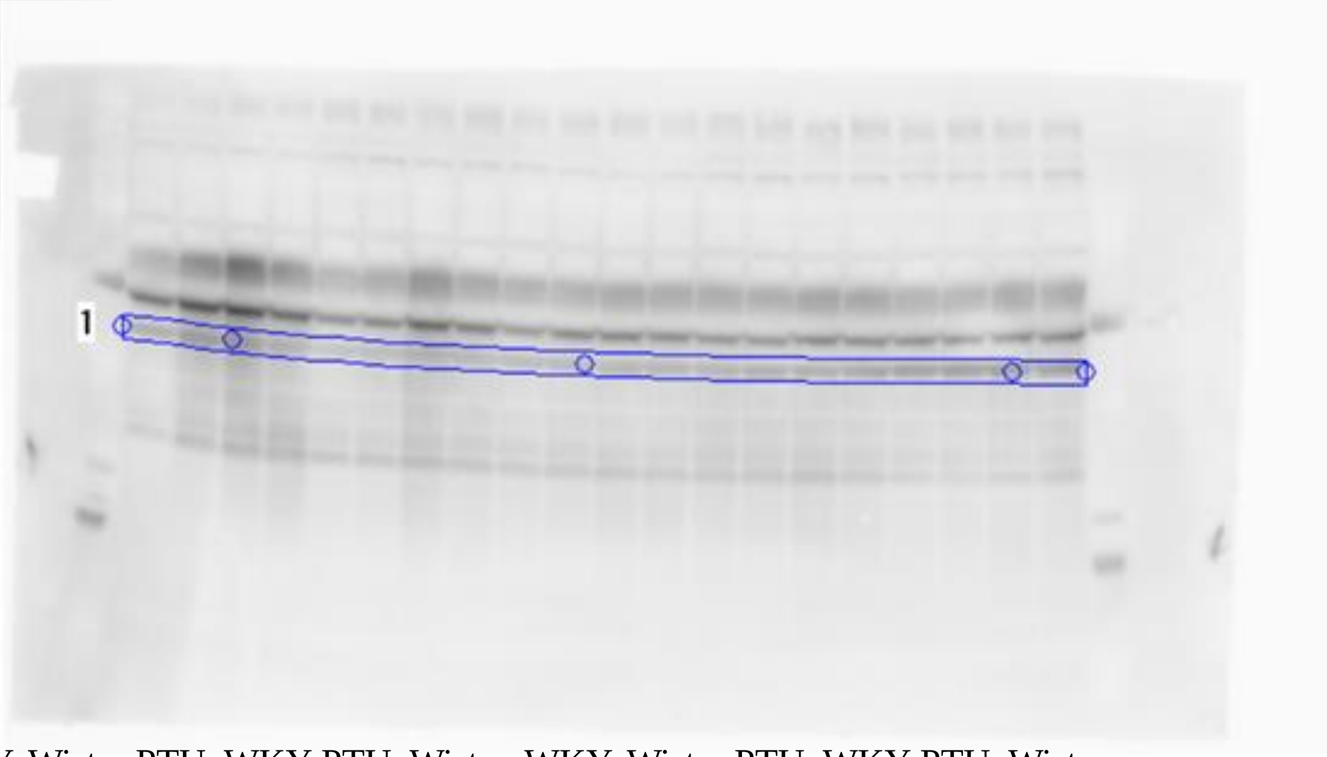

The bands from the left: Wistar, WKY, Wistar PTU, WKY PTU, Wistar, WKY, Wistar PTU, WKY PTU, Wistar, WKY, Wistar PTU, WKY PTU, Wistar, WKY, Wistar PTU, WKY PTU, Wistar, WKY, Wistar PTU, WKY PTU

p-CREB

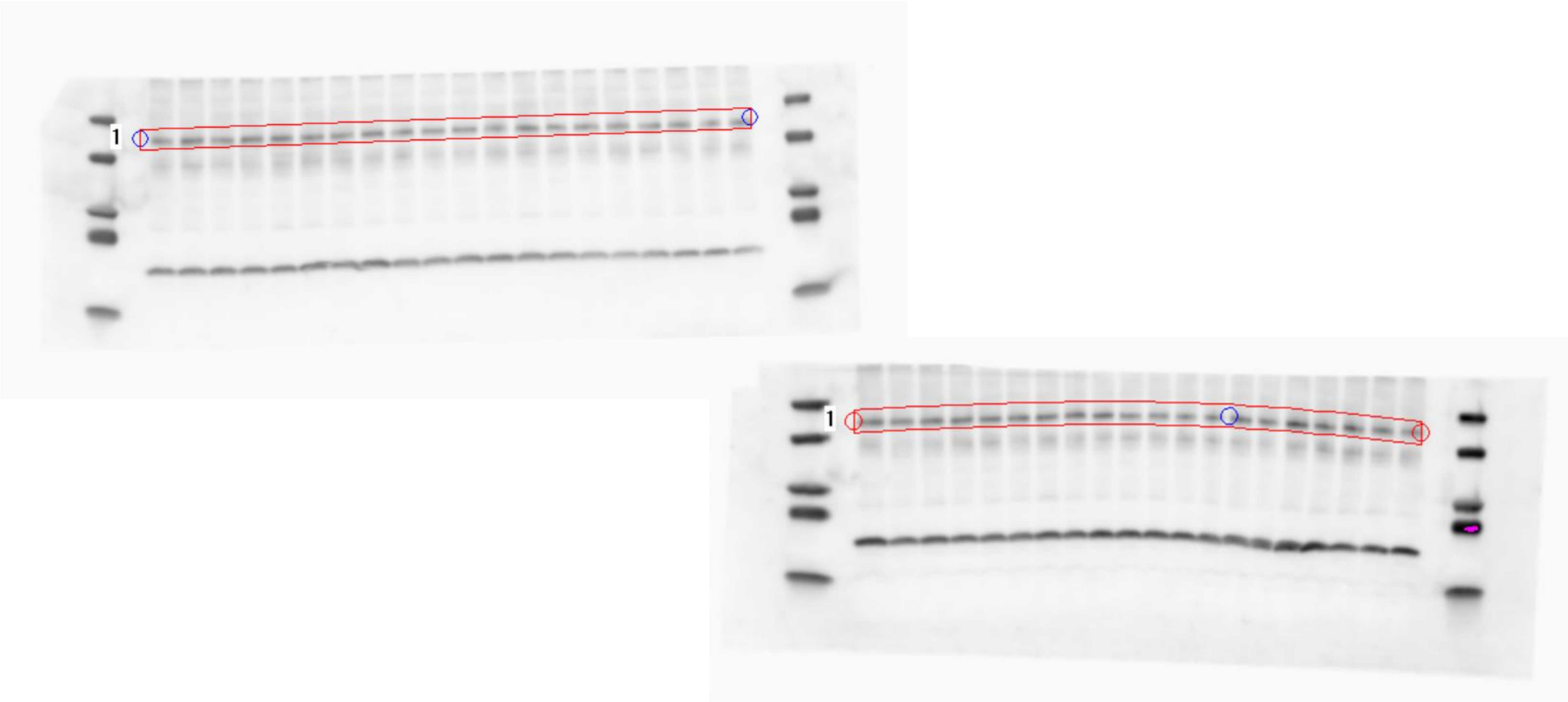

The bands from the left: Wistar, WKY, Wistar PTU, WKY PTU, Wistar, WKY, Wistar PTU, WKY PTU, Wistar, WKY, Wistar PTU, WKY PTU, Wistar, WKY, Wistar PTU, WKY PTU, Wistar, WKY, Wistar PTU, WKY PTU

CREB

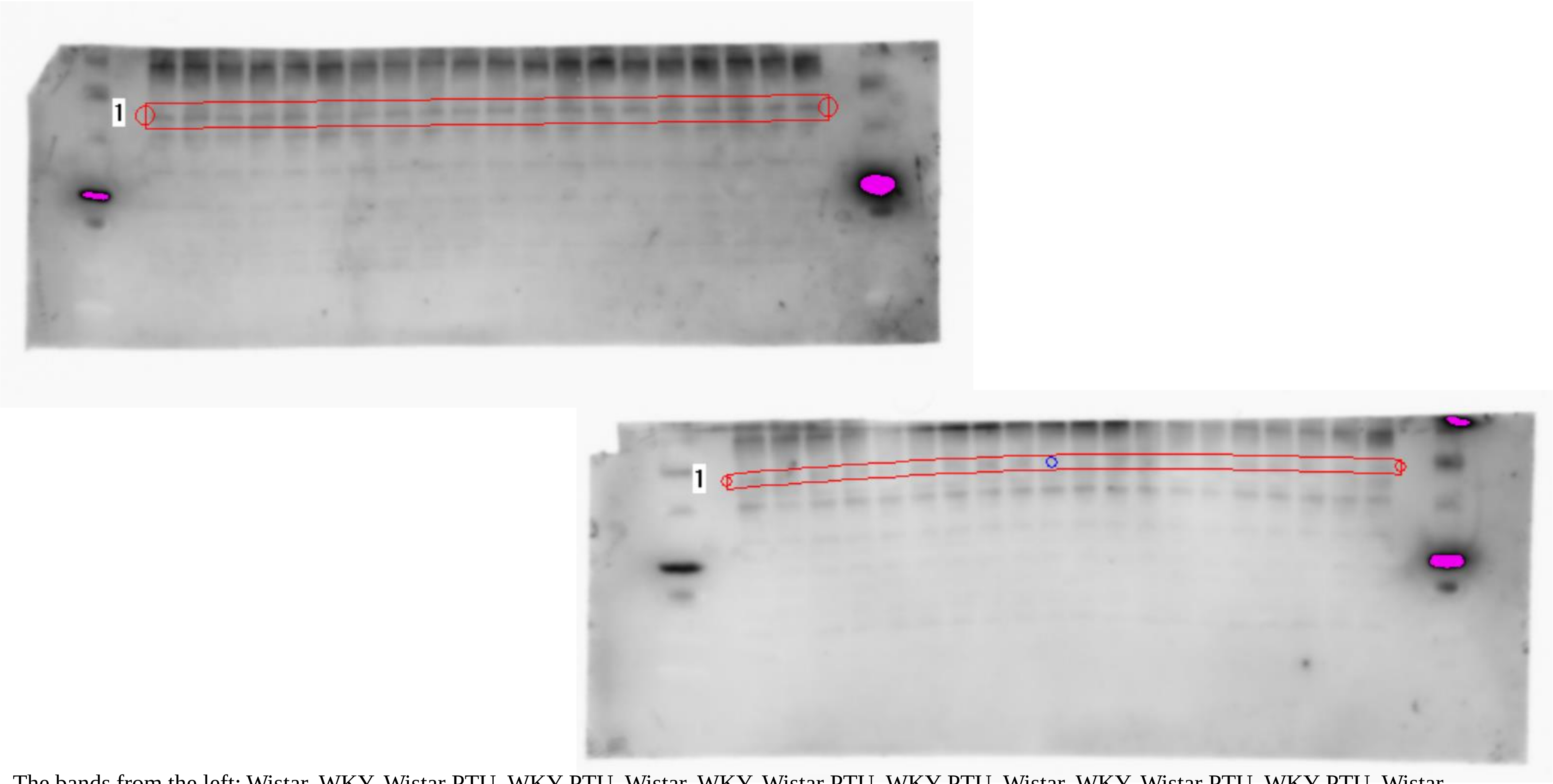

The bands from the left: Wistar, WKY, Wistar PTU, WKY PTU, Wistar, WKY, Wistar PTU, WKY PTU, Wistar, WKY, Wistar PTU, WKY PTU, Wistar, WKY, Wistar PTU, WKY PTU, Wistar, WKY, Wistar PTU, WKY PTU

β-actin (as loading control) to p-CREB and CREB proteins

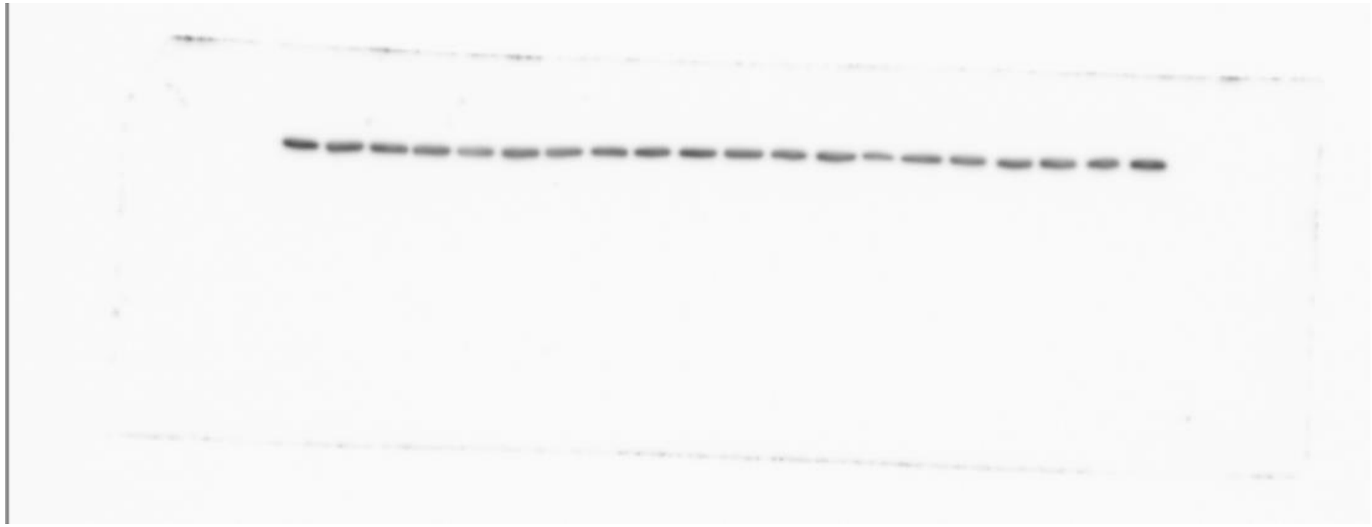

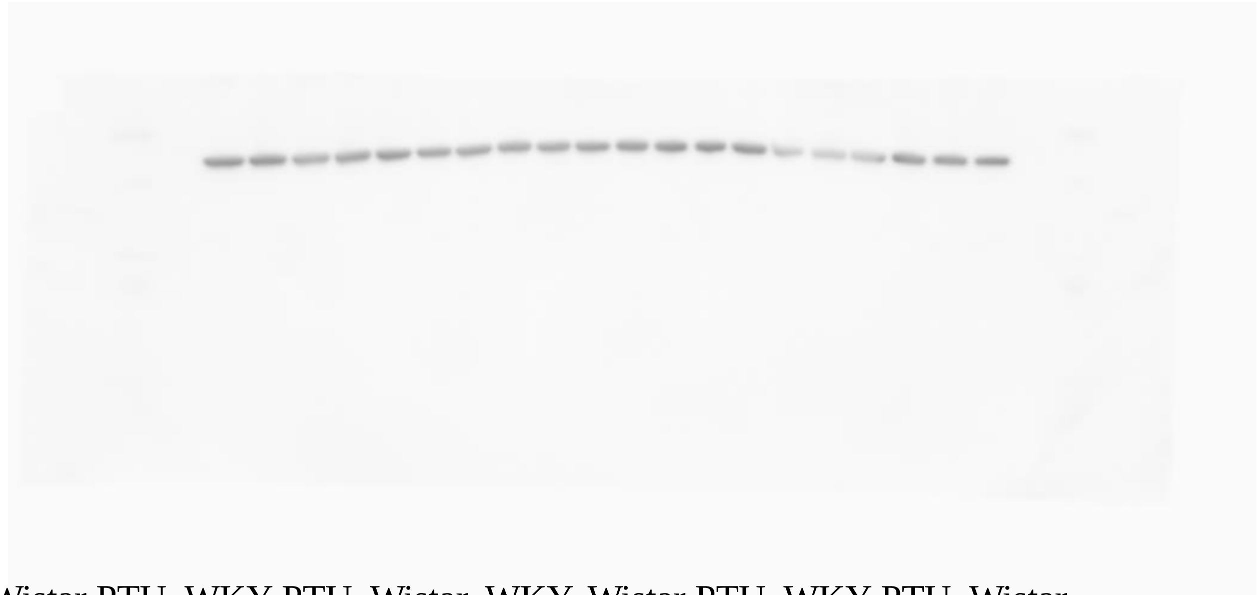

The bands from the left: Wistar, WKY, Wistar PTU, WKY PTU, Wistar, WKY, Wistar PTU, WKY PTU, Wistar, WKY, Wistar PTU, WKY PTU, Wistar, WKY, Wistar PTU, WKY PTU, Wistar, WKY, Wistar PTU, WKY PTU

p-ERK1

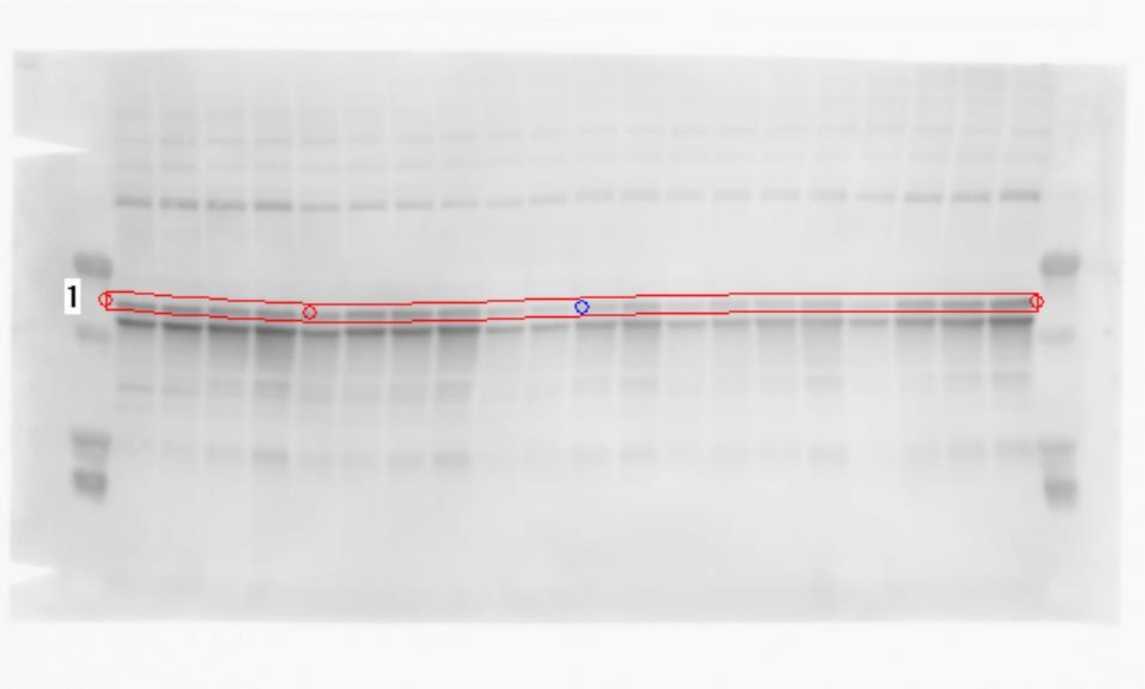

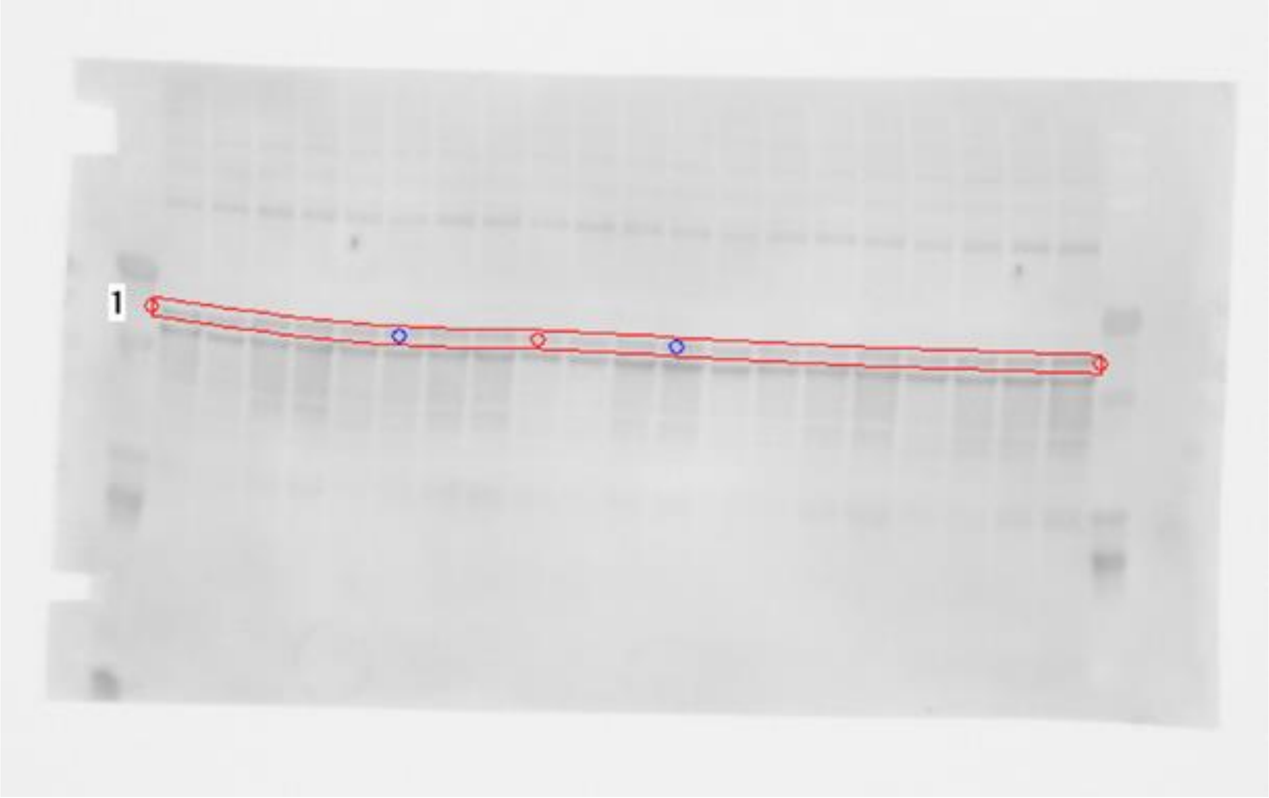

The bands from the left: Wistar, WKY, Wistar PTU, WKY PTU, Wistar, WKY, Wistar PTU, WKY PTU, Wistar, WKY, Wistar PTU, WKY PTU, Wistar, WKY, Wistar PTU, WKY PTU, Wistar, WKY, Wistar PTU, WKY PTU

ERK1

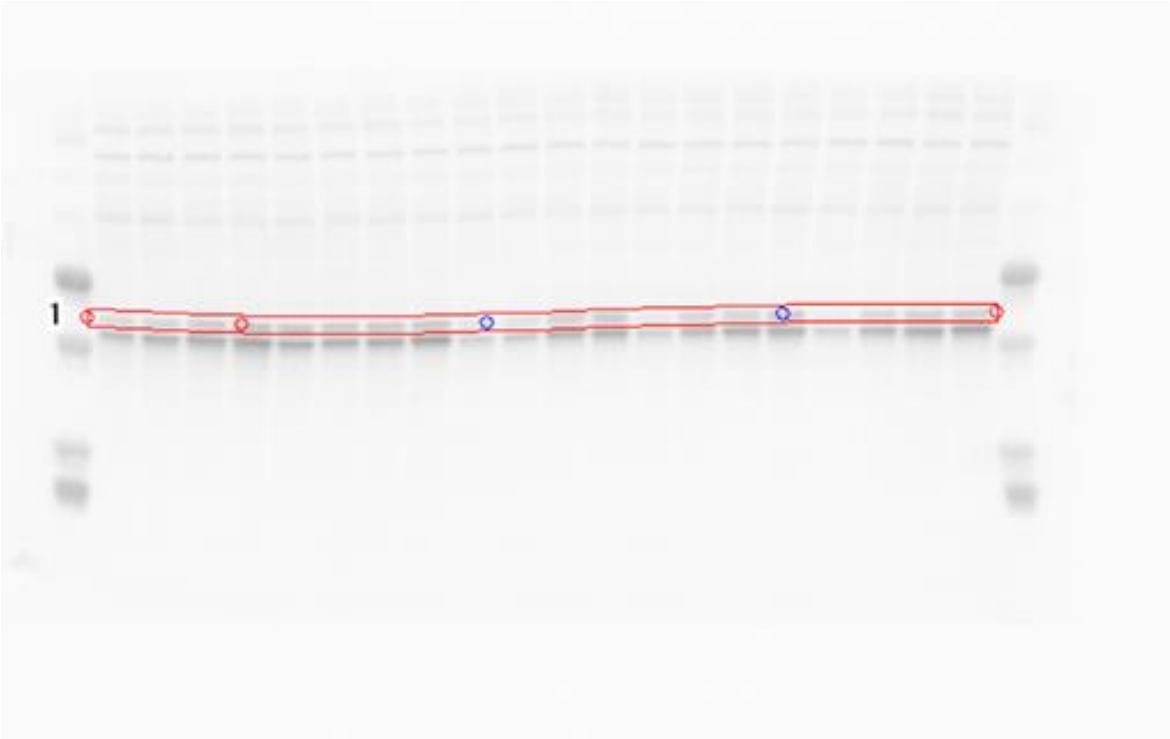

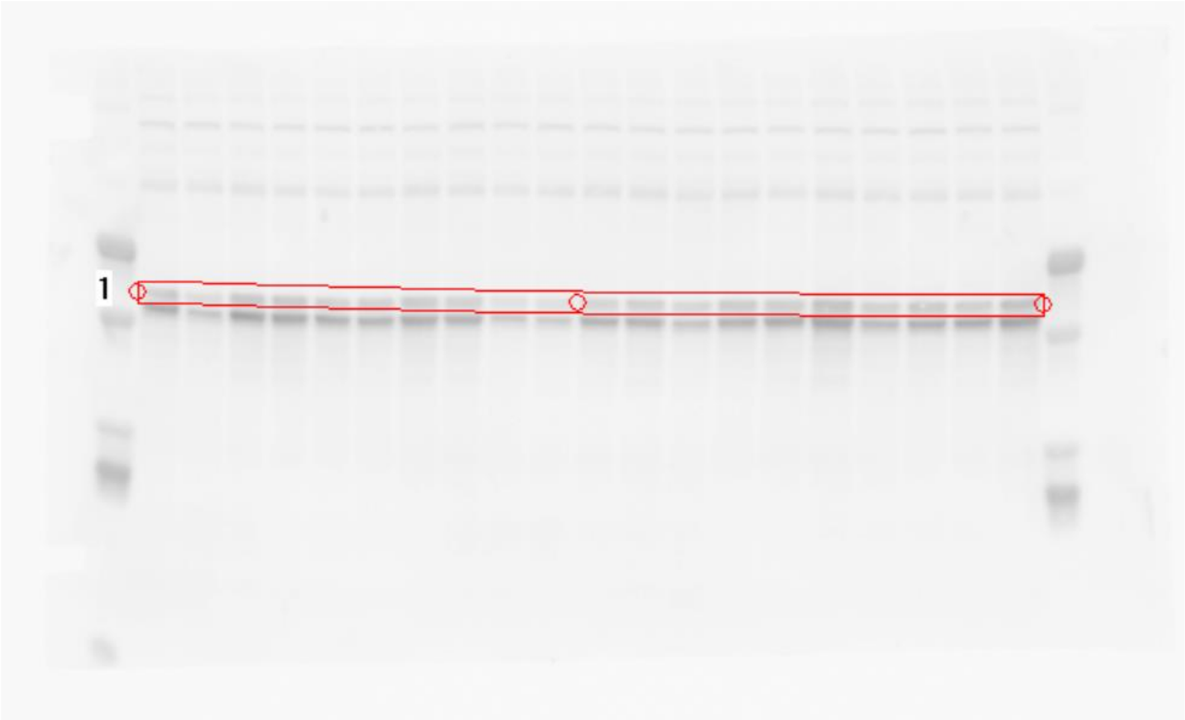

The bands from the left: Wistar, WKY, Wistar PTU, WKY PTU, Wistar, WKY, Wistar PTU, WKY PTU, Wistar, WKY, Wistar PTU, WKY PTU, Wistar, WKY, Wistar PTU, WKY PTU, Wistar, WKY, Wistar PTU, WKY PTU

p-ERK2

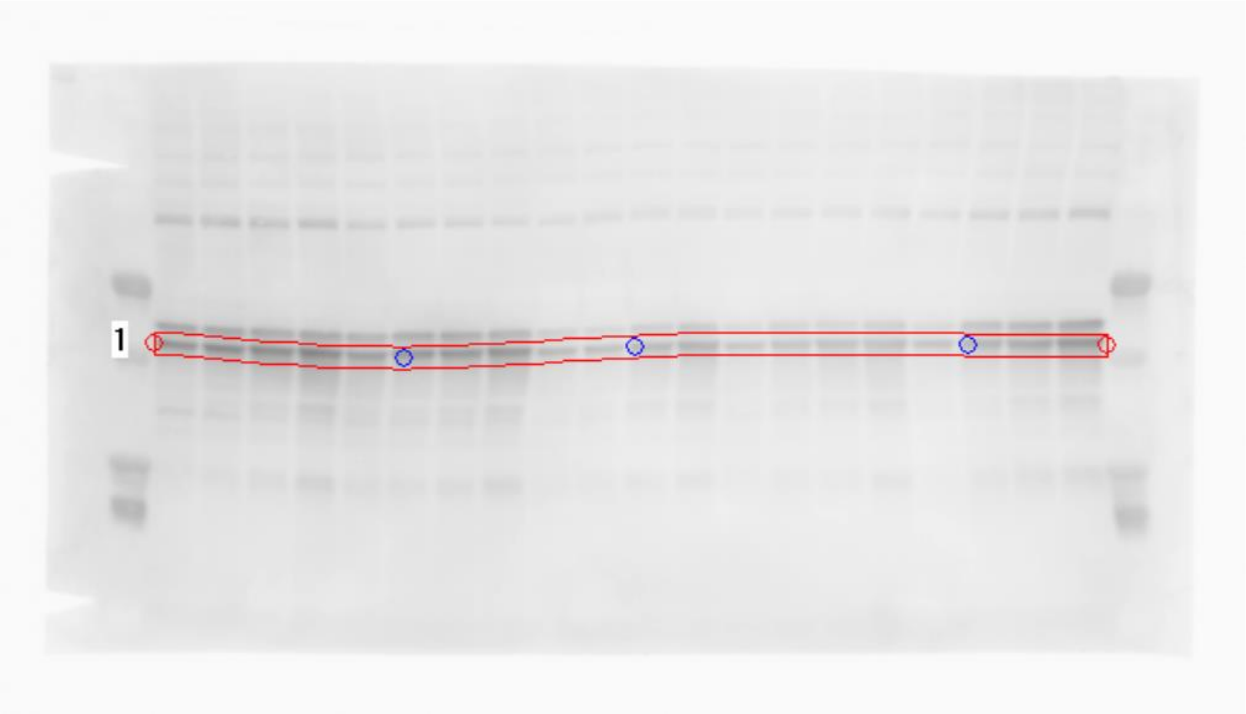

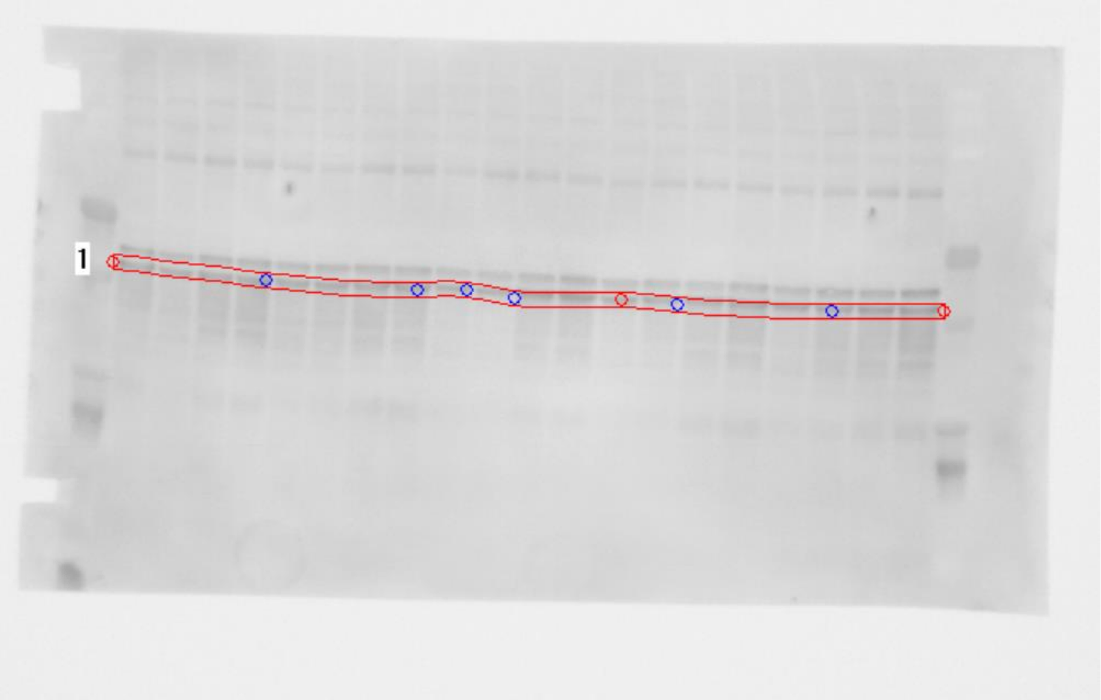

The bands from the left: Wistar, WKY, Wistar PTU, WKY PTU, Wistar, WKY, Wistar PTU, WKY PTU, Wistar, WKY, Wistar PTU, WKY PTU, Wistar, WKY, Wistar PTU, WKY PTU, Wistar, WKY, Wistar PTU, WKY PTU

ERK2

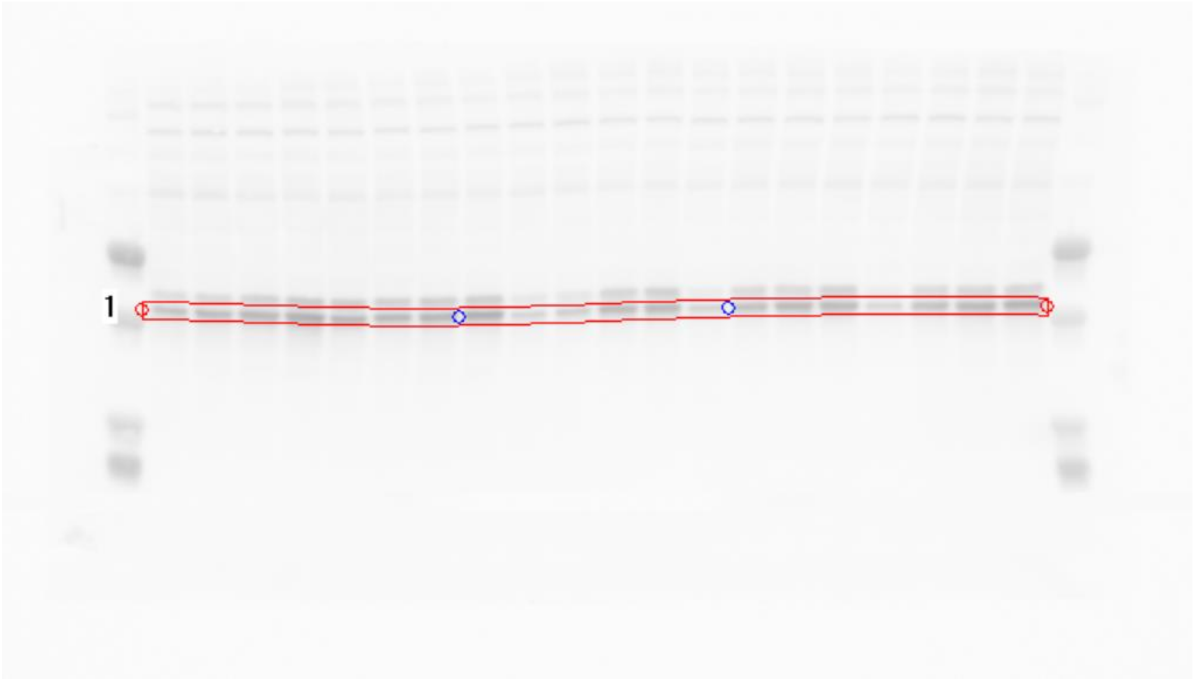

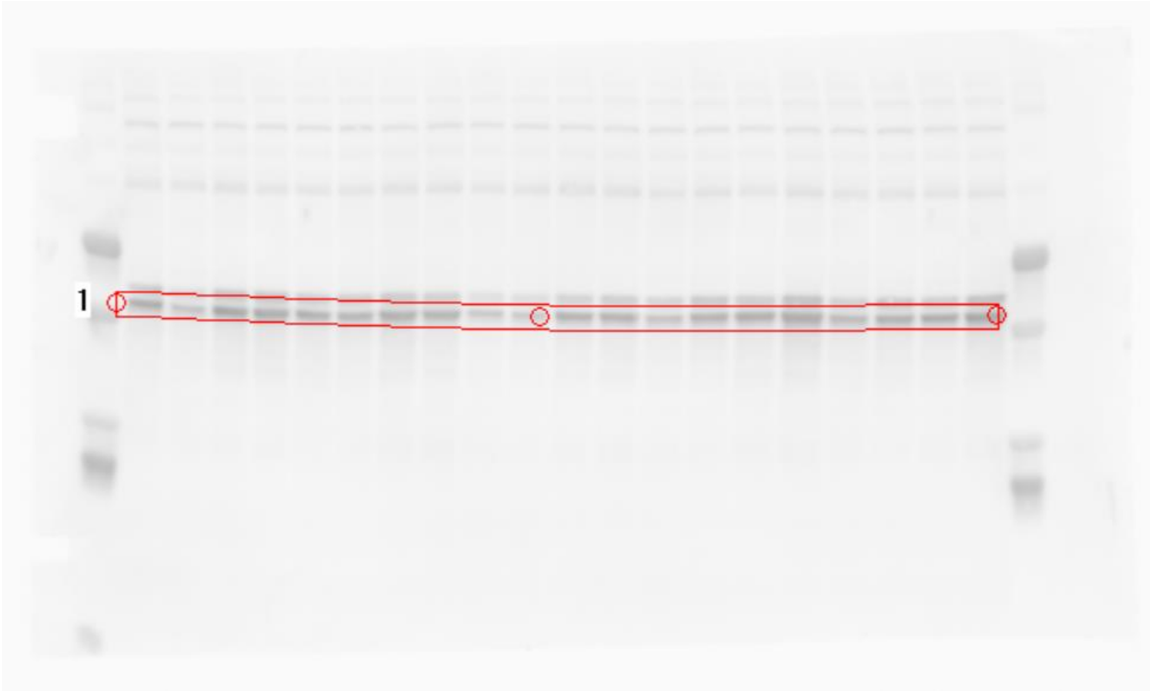

The bands from the left: Wistar, WKY, Wistar PTU, WKY PTU, Wistar, WKY, Wistar PTU, WKY PTU, Wistar, WKY, Wistar PTU, WKY PTU, Wistar, WKY, Wistar PTU, WKY PTU, Wistar, WKY, Wistar PTU, WKY PTU

β-actin (as loading control) to p-ERK1, p-ERK2 and total ERK-1 and ERK-2 proteins

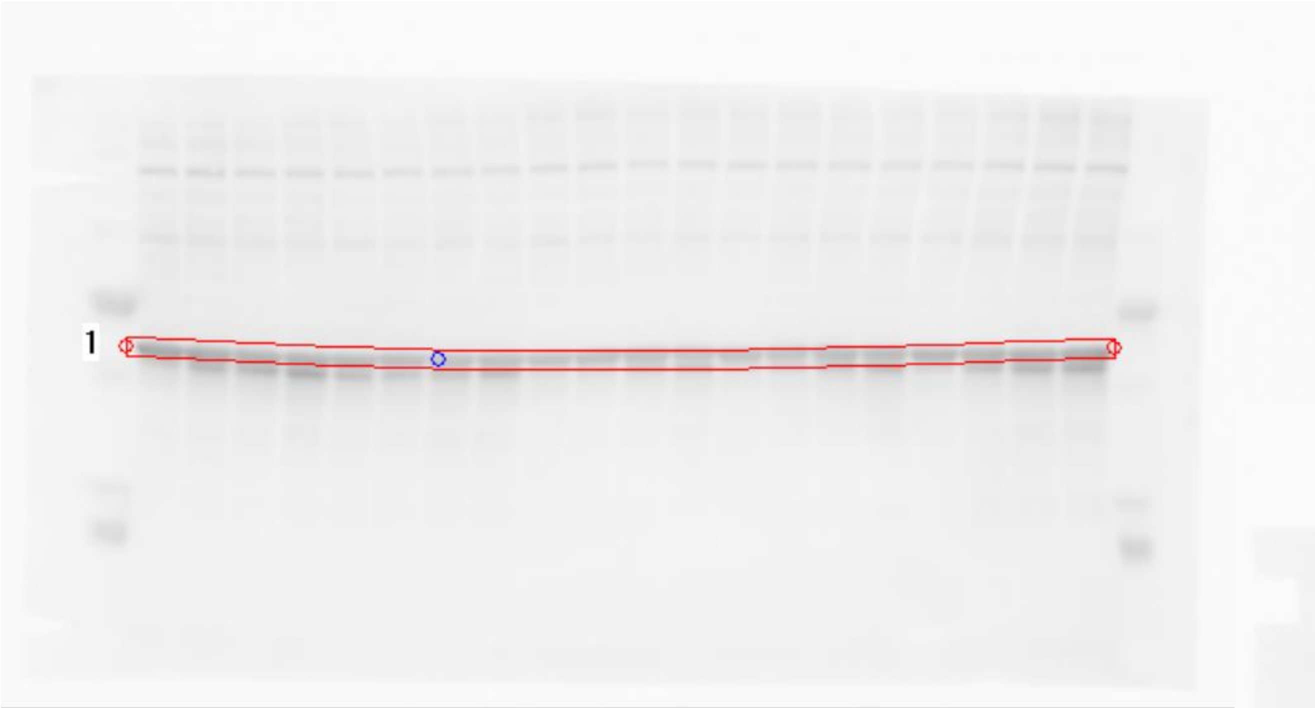

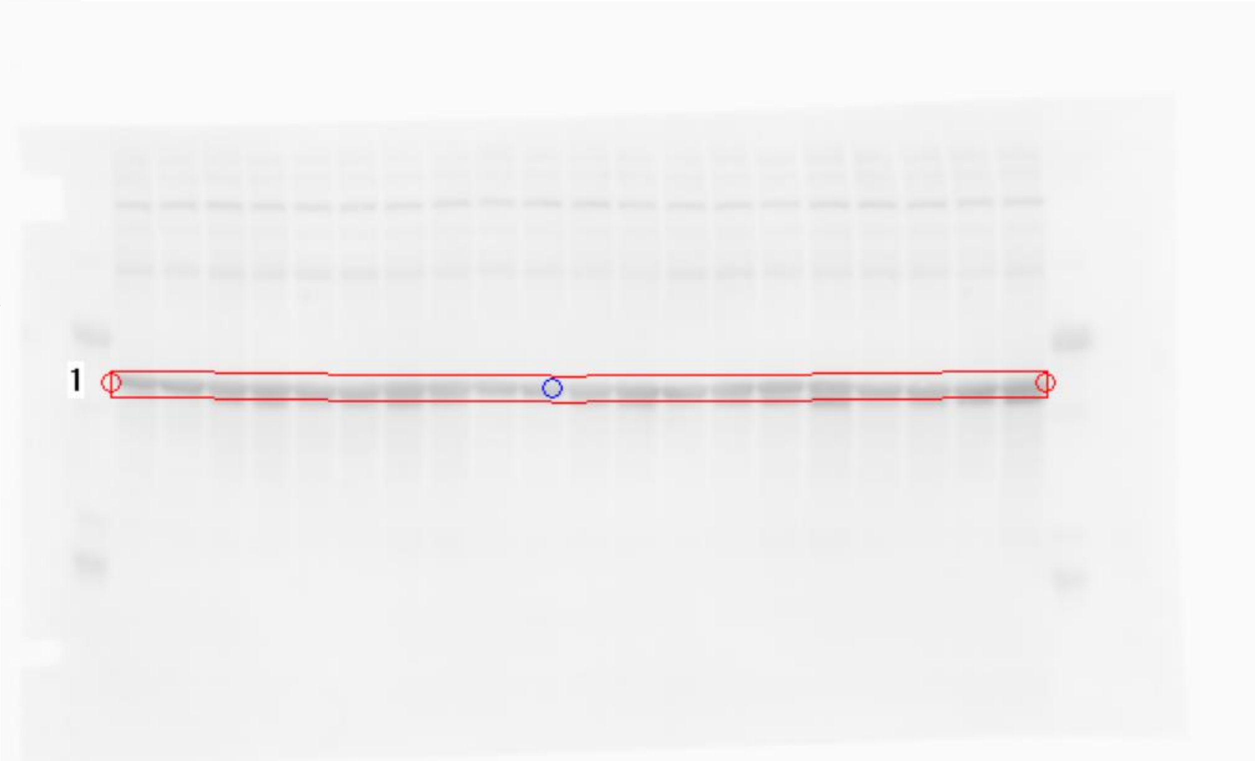

The bands from the left: Wistar, WKY, Wistar PTU, WKY PTU, Wistar, WKY, Wistar PTU, WKY PTU, Wistar, WKY, Wistar PTU, WKY PTU, Wistar, WKY, Wistar PTU, WKY PTU, Wistar, WKY, Wistar PTU, WKY PTU

not all samples (presented on pages1-19) were analyzed (poor quality or outliers were not analyzed)
